# Triple Redox–Enabled High‐Entropy Metal–Organic Coordination Driving High‐Performance Aqueous Zinc–Ion Batteries

**DOI:** 10.1002/advs.202511748

**Published:** 2025-08-12

**Authors:** Qian Li, Yanfei Zhang, Ziming Qiu, Meng Du, Shengxu Wei, Yiwen Liu, Wanchang Feng, Huan Pang

**Affiliations:** ^1^ School of Chemistry and Chemical Engineering Yangzhou University Yangzhou Jiangsu 225002 P. R. China; ^2^ School of Chemistry and Chemical Engineering Chongqing University of Science and Technology Chongqing 401331 P. R. China; ^3^ School of Environmental Science Nanjing Xiaozhuang University Nanjing Jiangsu 211171 P. R. China

**Keywords:** aqueous batteries, high entropy, practical application, reaction mechanism, triple redox–enabled

## Abstract

High entropy (HE) nanomaterials offer a promising strategy for advancing aqueous zinc–ion batteries (AZIBs). In this study, 1,4‐dihydroxyanthraquinone (1,4‐DHAQ) is employed as an organic ligand to coordinate with multiple metal ions, forming a series of binary to quinary high‐entropy (HE) nanomaterials. All synthesized materials exhibit a layered flower cluster structure. Among them, MnCoNiFeCu‐1,4‐DHAQ (denoted as HE‐1,4‐DHAQ) demonstrates the largest specific surface area and pore volume, along with superior electronic conductivity. HE‐1,4‐DHAQ delivers outstanding electrochemical performance, maintaining a high specific capacity of 222.6 mAh·g^−1^ after 150 cycles at a current density of 0.3 A·g^−1^. A combination of in situ powder X‐ray diffraction, ultraviolet and visible spectrophotometry, and Fourier transform infrared, together with ex situ X‐ray photoelectron spectroscopy and elemental mapping, is employed to comprehensively elucidate the structural evolution and reaction mechanisms during cycling. The results reveal that HE‐1,4‐DHAQ exhibits excellent structural stability and a highly reversible Zn^2+^ insertion/extraction mechanism. Moreover, the involvement of three active centers, namely the ─OH groups on the ligand, Mn, and Cu, is confirmed. Notably, HE‐1,4‐DHAQ has been applied as a cathode in soft‐pack batteries, gel electrolyte, and screen‐printed devices, demonstrating strong potential for energy storage and flexible electronics.

## Graphical abstract

Four nanomaterials, from binary to high‐entropy (HE), are synthesized using π‐conjugated 1,4‐DHAQ ligand for aqueous zinc–ion batteries. HE‐1,4‐DHAQ shows high specific capacity and cycling stability. In situ and ex situ analyses reveal a stable framework and reversible Zn^2+^ insertion/extraction. It demonstrates strong practical potential in soft–pack batteries, gel electrolyte devices, and screen‐printing systems.

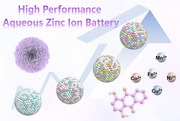

## 1 Introduction

High‐efficiency and safe storage technology is a critical component and core challenge in achieving a sustainable energy system.^[^ ^1^, ^2^, ^3^ ^]^ In recent years, aqueous zinc–ion batteries (AZIBs) have emerged as promising candidates for next‐generation large‐scale energy storage technologies, owing to their intrinsic safety derived from water‐based electrolytes, the low cost and abundant availability of Zn metal anodes, and their relatively high theoretical energy density.^[^ ^4^, ^5^, ^6^, ^7^ ^]^ Currently, the cathode materials for AZIBs primarily include Mn‐based oxides, V‐based compounds, and organic redox–active species.^[^ ^8^, ^9^, ^10^ ^]^ However, each of these conventional materials faces inherent limitations: Mn‐based oxides, despite their high theoretical capacity, often suffer from capacity fading due to the Jahn–Teller effect and structural collapse during cycling;^[^ ^11^ ^]^ V‐based compounds exhibit excellent electrochemical performance but are hindered by high cost and potential heavy metal toxicity;^[^ ^12^ ^]^ organic cathode materials, while offering tunable molecular structures and renewability, often experience severe dissolution of small‐molecule species in aqueous electrolytes, significantly compromising their cycling stability and rate capability.^[^ ^13^ ^]^ To enhance the overall performance of cathode materials, researchers have explored strategies such as superstructure^[^ ^14^, ^15^ ^]^ and electrolyte system optimization.^[^ ^16^, ^17^ ^]^ Nevertheless, the increased complexity of synthesis, higher production costs, and limited effectiveness of these approaches continue to impede the practical deployment of AZIBs.

Under this context, high‐entropy (HE) nanomaterials, benefiting from the entropy effect generated by the synergistic interaction of multiple components, exhibit exceptional crystal structure stability, ion diffusion regulation capabilities, and chemical compositional diversity.^[^ ^18^, ^19^, ^20^, ^21^ ^]^ These attributes position them at the forefront of electrode material research. Introducing the HE concepts into cathode design for AZIBs enhances the entropy stability of the system through the random distribution of multimetallic centers.^[^ ^22^, ^23^ ^]^ This approach can effectively suppress unfavorable phase transitions from a thermodynamic perspective, while also optimizing the transport pathways of electrons and Zn^2+^ from a kinetic standpoint. The synergistic effect of multiple active sites further broadens the electrochemical reaction platform, thereby achieving high capacity without compromising cycle life‐realizing both high capacity and stability. The metal–organic coordination (MOCs), featuring an ordered coordination structure composed of metal centers and organic ligands, offers a tunable chemical environment and a controllable synthesis process, thereby providing an ideal platform for the construction of HE electrode materials.^[^ ^24^, ^25^ ^]^ By modulating the metal centers, MOCs can be engineered with HE characteristics while also providing excellent structural tunability.

In this study, 1,4‐dihydroxyanthraquinone (1,4‐DHAQ), featuring an extended π‐conjugated framework, was chosen as the ligand. Five transition metals with similar ionic radii, low toxicity, and low cost (Mn^2+^, Co^2+^, Ni^2+^, Fe^2+^, and Cu^2+^) were selected and used to synthesize a series of binary to quinary metal–organic coordination compounds via a hydrothermal method. These materials were then investigated as cathodes for AZIBs. Among them, the HE nanomaterial MnCoNiFeCu‐1,4‐DHAQ (referred to as HE‐1,4‐DHAQ) exhibits synergistic redox reactions involving multiple active centers during the electrochemical process, including Mn centers, Cu centers, and ─OH groups on the ligand redox activity, accompanied by reversible Zn^2+^ and H^+^ insertion/extraction, demonstrating excellent zinc storage performance. Specifically, the HE‐1,4‐DHAQ electrode retained a high specific capacity of ≈222.6 mAh·g^−1^ after 150 cycles at a current density of 0.3 A·g^−1^. Moreover, it delivered excellent cycling stability, maintaining consistent performance over 3000 cycles at 1.0 A·g^−1^. The charge–discharge mechanism of HE‐1,4‐DHAQ was systematically elucidated through a combination of in situ powder X‐ray diffraction (XRD), ultraviolet and visible spectrophotometry (UV–vis), and Fourier transform infrared (FTIR) spectral, as well as ex situ X‐ray photoelectron spectroscopy (XPS), and elemental mapping characterization. Importantly, when applied as the cathode in soft‐pack battery, gel electrolyte devices, and screen‐printed devices, HE‐1,4‐DHAQ exhibited excellent mechanical stability and wearable characteristics, highlighting its potential for practical applications. In summary, the HE nanomaterials developed in this work offer a novel design strategy and technological platform for advancing AZIB cathode materials.

## 2 Results and Discussion

**Figure 1:**
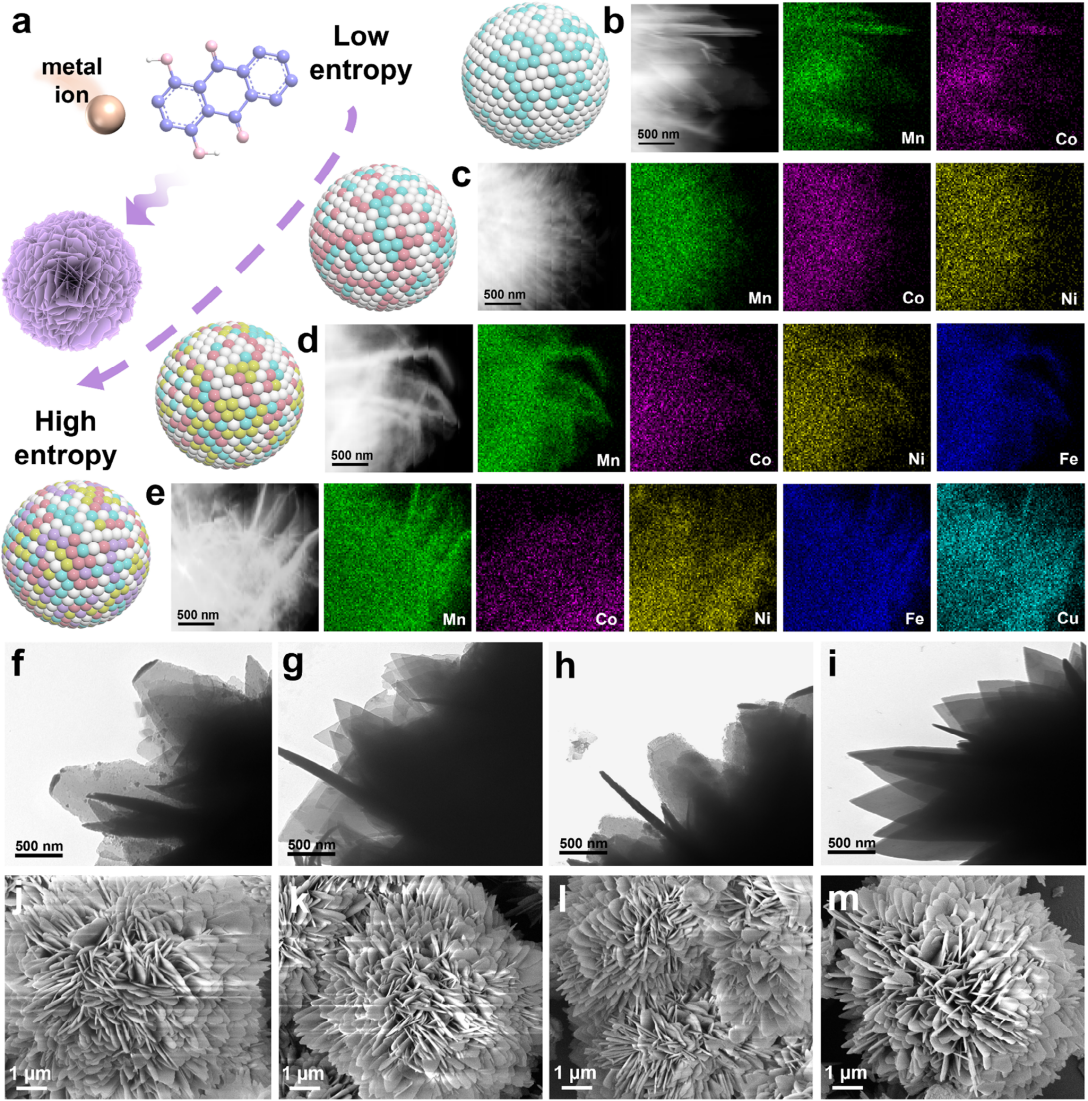
a) Schematic illustration of the synthesis route for binary to HE nanomaterials. The elemental mapping images of b) MnCo‐1,4‐DHAQ, c) MnCoNi‐1,4‐DHAQ, d) MnCoNiFe‐1,4‐DHAQ, and e) HE‐1,4‐DHAQ, respectively. TEM and SEM images of f,j) MnCo‐1,4‐DHAQ, g,k) MnCoNi‐1,4‐DHAQ, h,l) MnCoNiFe‐1,4‐DHAQ, and i,m) HE‐1,4‐DHAQ, respectively.

Five metal cations (Mn^2+^, Co^2+^, Ni^2+^, Fe^2+^, and Cu^2+^) were used as metal sources, and 1,4‐DHAQ, which conjugated π‐electron and quinone‐based redox activity, served as the ligand. Using DMF/ethanol as the solvent and a hydrothermal synthesis route, binary (MnCo‐1,4‐DHAQ), ternary (MnCoNi‐1,4‐DHAQ), quaternary (MnCoNiFe‐1,4‐DHAQ), and HE‐1,4‐DHAQ nanomaterials were successfully prepared (**Figure** **1**a). The detailed synthetic procedures are provided in the Supporting Information. Elemental mapping and energy‐dispersive X‐ray (EDX) imaging were then employed to systematically examine the distribution of each element in the four materials. The results demonstrate that all five metal elements are uniformly distributed throughout each nanomaterial (Figure 1b–e; Figures S1–S4, Supporting Information). Transmission electron microscopy (TEM) revealed that all the synthesized materials exhibit thin‐layered structures, which can increase the electrode–electrolyte interfacial area and thereby promote electrochemical reactions (Figure 1f–i; Figure S5, Supporting Information).^[^ ^26^, ^27^ ^]^ Meanwhile, scanning electron microscopy (SEM) images show that the nanomaterials possess a 3D layered nanoflower morphology, which not only provides more active sites but also effectively shortens the diffusion path of Zn^2+^ within the material. This structural feature facilitates faster Zn^2+^ insertion/extraction and enhances cycling stability (Figure 1j–m).^[^ ^28^, ^29^ ^]^

**Figure 2:**
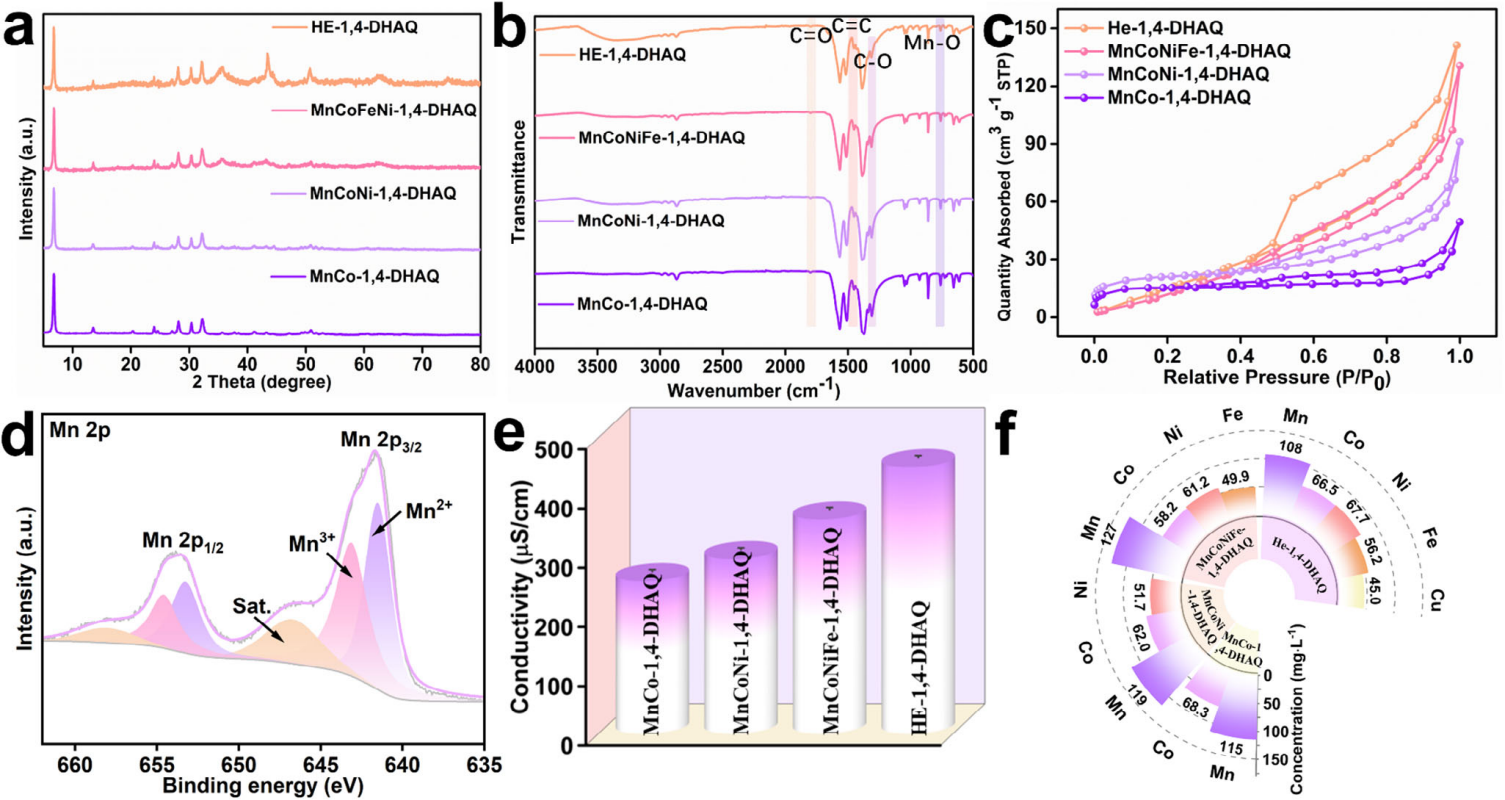
a) XRD pattern, b) FTIR spectra and c) N_2_ adsorption–desorption isotherms of nanomaterials. d) XPS spectrum of Mn 2p of HE‐1,4‐DHAQ. e) Electrical conductivity of nanomaterials. f) Metal ions concentration of nanomaterials.

XRD analysis revealed that the series of materials synthesized from binary to HE compositions exhibit similar diffraction peak positions, which is consistent with previously reported results (**Figure** **2**a).^[^ ^30^ ^]^ Compared with the binary to quaternary materials, the HE‐1,4‐DHAQ sample synthesized with the addition of Cu^2+^ shows noticeable differences in diffraction peak intensity, likely due to increased structural defects induced by the incorporation of Cu^2+^. FTIR spectroscopy was used to analyze the composition of the four synthesized nanomaterials. The signals observed at 760, 1313, 1454, and 1797 cm^−1^ correspond to the stretching vibrations of Mn─O, C─O, C═C, and C═O bonds, respectively, confirming the successful coordination between the metal ions and the 1,4‐DHAQ ligand (Figure 2b). The N_2_ adsorption–desorption isotherm was used to comparatively analyze the specific surface areas and pore size distributions of the different materials. The results show that both the specific surface area and average pore size increase significantly as the metal composition progresses from binary to HE systems (Figure 2c; Figures S6 and S7 and Table S1, Supporting Information).^[^ ^31^ ^]^ This trend can be mainly attributed to two factors: the synergistic effect of multiple metal species introduces more structural defects during the hydrothermal synthesis process, while the HE effects help stabilize and maintain a hierarchical porous network. The combined influence of these factors endows the HE nanomaterial with a larger specific surface area and broader pore size distribution compared to the binary counterpart. XPS was employed to analyze the elemental composition and valence states of the synthesized nanomaterials (Figure 2d; Figures S8–S11, Supporting Information). The Mn 2p spectrum displays the characteristic Mn 2p_3/2_ and Mn 2p_1/2_ peaks, indicating that Mn primarily exists in the +2 state.^[^ ^32^ ^]^ Similarly, the Co 2p spectrum shows distinct Co 2p_3/2_ and Co 2p_1/2_ peaks. The Ni 2p spectrum reveals a mixed +2/+3 oxidation state, with the +2 state being predominant. The Fe 2p spectrum also indicates the coexistence of +2 and +3 states. In the Cu 2p spectrum, Cu is mainly present in the +2 state. Figure 2e shows the electrical conductivity measurements of the nanomaterials. The results indicate that as the metal composition increases from binary systems to HE systems, the electrical conductivity of the materials improves significantly. This phenomenon can be attributed to the “cocktail effect” of multiple metal centers, which facilitates the migration of electrons and ions. In addition, the synergistic π‐conjugation between the 1,4‐DHAQ ligands and the d‐orbitals of various metal ions further enhances charge carrier transport. In addition, the elemental composition of the binary to HE nanomaterials were determined using inductively coupled plasma–optical emission spectrometry (ICP–OES) (Figure 2f; Table S2, Supporting Information). The results confirm that the elements are uniformly distributed within the materials, providing strong evidence for the successful synthesis of all four nanomaterials, particularly the effective construction of the HE composition.^[^ ^33^, ^34^ ^]^

**Figure 3:**
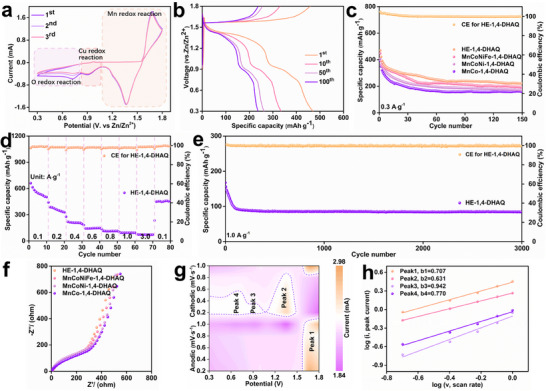
a) CV curves at a scan of 0.6 mV·s^−1^ of HE‐1,4‐DHAQ. b) Charge–discharge profiles of HE‐1,4‐DHAQ. c) Cycling performance of nanomaterials at 0.3 A·g^−1^. d) Rate performance of HE‐1,4‐DHAQ. e) Cycling performance of HE‐1,4‐DHAQ at 1.0 A·g^−1^. f) EIS spectra of nanomaterials. g) Contour plots of CV patterns and h) determination of the *b*‐values for HE‐1,4‐DHAQ, respectively.

The electrochemical performance of binary, ternary, quaternary, and HE nanomaterials for AZIBs was systematically investigated. The synthesized nanomaterials were subjected to cyclic voltammetry (CV) tests within a voltage window of 0.3–1.8  V at a scan rate of 0.6 mV·s^−1^. The CV curves of MnCo‐1,4‐DHAQ, MnCoNi‐1,4‐DHAQ, and MnCoNiFe‐1,4‐DHAQ exhibited a pair of redox peaks in the range of 0.3–0.8  V, corresponding to the redox activity of ─OH groups on the organic ligand.^[^ ^30^ ^]^ Additionally, another pair of redox peaks appeared in the range of 0.8–1.8  V, attributed to the reversible redox behavior of Mn species (Figure S12, Supporting Information).^[^ ^35^, ^36^ ^]^ In contrast, the CV curve of HE‐1,4‐DHAQ showed not only the redox peaks related to the ─OH groups and Mn within the voltage ranges but also an additional pair of peaks in the range of 0.8–1.1  V, corresponding to the reversible redox reaction of Cu (**Figure** **3**a).^[^ ^37^ ^]^ The presence of three voltage plateaus in the galvanostatic charge–discharge profiles was consistent with the three sets of redox peaks observed in the CV curves (Figure 3b; Figure S13, Supporting Information). Therefore, the HE‐1,4‐DHAQ cathode exhibits three distinct redox‐active centers during the charge–discharge process, namely the ─OH groups on the ligand, Mn, and Cu. Compared to the binary, ternary, and quaternary systems, this multicomponent synergy significantly increases the number of accessible redox‐active sites, thereby providing more reaction pathways to enhance the specific capacity of the electrode material. To investigate the synergistic effect of Zn^2+^ and H^+^ during the multi‐step intercalation process, electrochemical tests were performed on HE‐1,4‐DHAQ in H_2_SO_4_ electrolytes of varying concentrations (Figure S14, Supporting Information). The results show that although the specific capacity of HE‐1,4‐DHAQ increases with H_2_SO_4_ concentration, it still does not reach the level measured in the 2 m ZnSO_4_·7H_2_O (pH 3.6) electrolyte. This indicates that both Zn^2+^ and H^+^ participate in the electrochemical reaction.

The electrochemical performance of four nanomaterials was systematically evaluated. At a current density of 0.3 A·g^−1^, the specific capacities of MnCo‐1,4‐DHAQ, MnCoNi‐1,4‐DHAQ, MnCoNiFe‐1,4‐DHAQ, and HE‐1,4‐DHAQ after 150 cycles were 162.4, 174.2, 191.8, and 222.6 mAh·g^−1^, respectively, all of which are higher than that of Mn 1,4‐DHAQ. Notably, HE‐1,4‐DHAQ exhibited a high Coulombic efficiency of 99.8% (Figure 3c; Figures S15 and S16, Supporting Information). Additionally, compared to other reported nanomaterials, HE‐1,4‐DHAQ exhibits superior electrochemical performance (Figure S17, Supporting Information). Rate performance was tested across a wide current density range from 0.1 to 3.0 A·g^−1^. The specific capacities of HE‐1,4‐DHAQ at current densities of 0.1, 0.2, 0.4, 0.6, 0.8, 1.0, and 3.0 A·g^−1^ were 660.4, 386.7, 220.2, 141.3, 117.4, 94.8, and 78.2 mAh·g^−1^, respectively. When the current density was returned to 0.1 A·g^−1^, the capacity recovered to 450.6 mAh·g^−1^, significantly outperforming the binary, ternary, and quaternary counterparts (Figure 3d; Figure S18, Supporting Information). To evaluate long‐term cycling stability under high current, the four materials were subjected to extended cycling at 1.0 A·g^−1^ for 3000 cycles (Figure 3e; Figure S19, Supporting Information). All samples demonstrated excellent stability, with HE‐1,4‐DHAQ maintaining a specific capacity of 84.1 mAh·g^−1^ after 3000 cycles. In electrochemical impedance spectroscopy (EIS) spectra, the high‐frequency semicircle corresponds to the charge‐transfer resistance (R*_ct_*), whereas the low‐frequency inclined line reflects the Zn^2+^ diffusion kinetics. EIS revealed that HE‐1,4‐DHAQ possessed the lowest R*_ct_*, indicating that entropy engineering significantly enhanced electrode kinetics (Figure 3f).^[^ ^38^ ^]^ Given its outstanding specific capacity and stability, the kinetic behavior of HE‐1,4‐DHAQ was further investigated. CV curves recorded at scan rates ranging from 0.2 to 1.0 mV·s^−1^ showed that the peak shapes remained largely unchanged with increasing scan rate, indicating high electrochemical stability (Figure 3g; Figure S20, Supporting Information). The peak current (*i*) and scan rate (*v*) followed the relationship *i = av^b^*, and the calculated *b*‐values for the four redox peaks ranged between 0.5 and 1.0, suggesting that the charge storage process is governed by a combination of ion diffusion and capacitive behavior (Figure 3h).^[^ ^39^, ^40^ ^]^

Based on the above results, the excellent electrochemical performance of HE‐1,4‐DHAQ can be attributed to the following factors: First, the presence of three redox‐active centers, namely the ─OH groups on the ligand, Mn, and Cu, provides abundant active sites for electrochemical reactions. Second, the HE structures composed of five transition metals (Mn, Fe, Co, Ni, and Cu) enables atomic‐level mixing, which generates a “cocktail effect” that enhances electron transport and synergistic catalytic activity. Third, the significantly increased specific surface area and pore volume improve electrolyte wettability and facilitate Zn^2+^ diffusion. Finally, the HE designs effectively suppresses structural collapse, greatly enhancing cycling stability. These combined advantages, including multiple redox centers, favorable kinetics, and robust structural integrity, contribute to the outstanding performance of HE‐1,4‐DHAQ in AZIBs.

**Figure 4:**
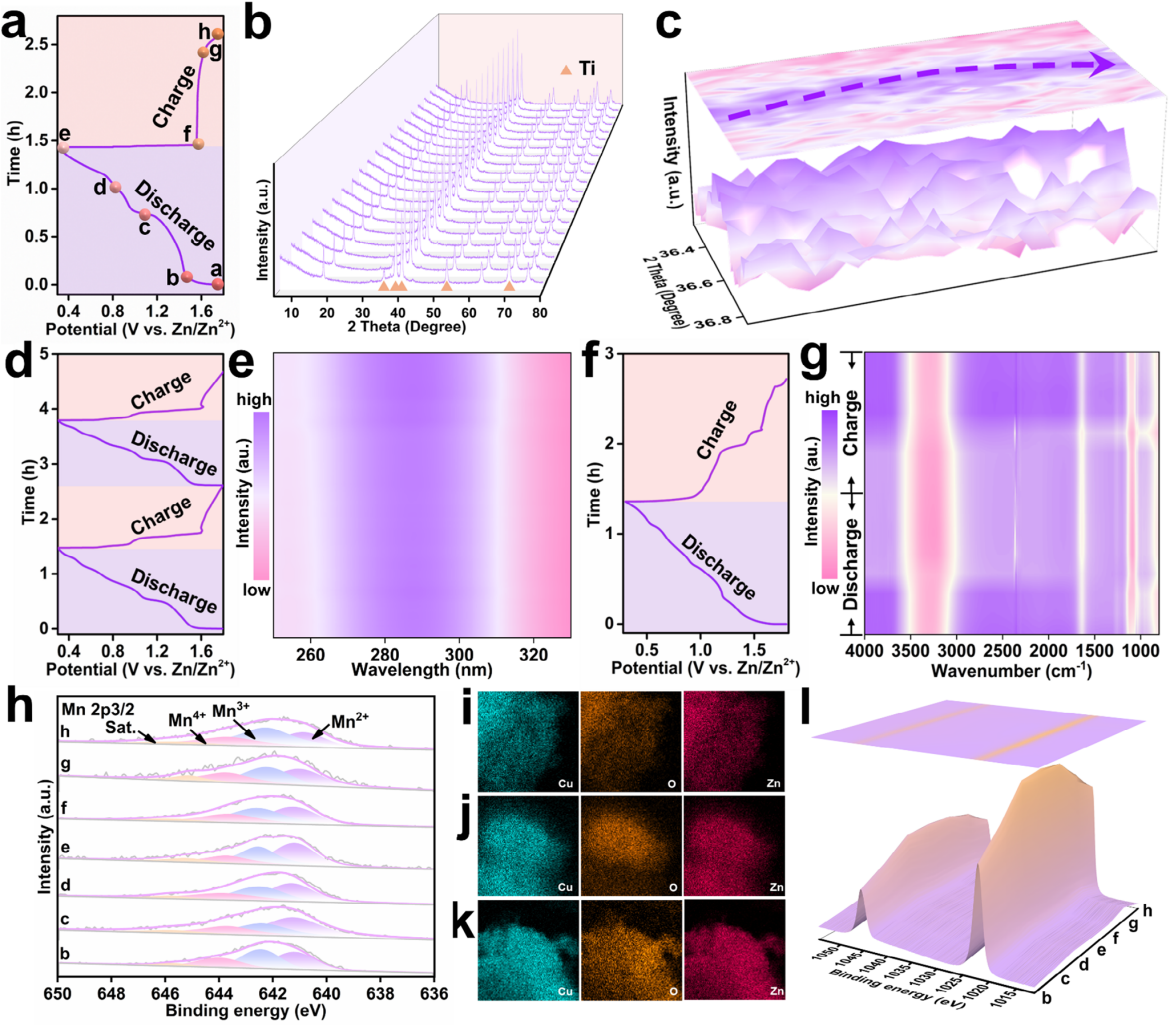
a–c) In situ XRD patterns, d,e) in situ UV–vis spectroscopy, and f,g) in situ FTIR spectra of HE‐1,4‐DHAQ during charge–discharge cycling, respectively. (In the image, the signal strength is represented by colors from strong too weak as purple, white, and pink, respectively.) h) Ex situ XPS spectra of Mn 2p of HE‐1,4‐DHAQ at different charge–discharge. Ex situ mapping images of HE‐1,4‐DHAQ at discharge to i) point c (1.1 V), j) point d (0.8 V), and k) point e (0.3 V), respectively. l) Ex situ XPS spectra of Zn 2p of HE‐1,4‐DHAQ at different charge–discharge.

To comprehensively investigate the energy storage mechanism of HE‐1,4‐DHAQ in AZIBs, this study employed a combination of in situ XRD, UV–vis, FTIR, and ex situ XPS and elemental mapping techniques. These multi‐dimensional characterizations enabled a detailed and systematic elucidation of the structural evolution and electrochemical behavior of HE‐1,4‐DHAQ during the charge–discharge process. In situ XRD results showed that, apart from a slight reversible shift of the 36.55° peak to a lower angle during discharge and its return to the original position upon charging, all other diffraction peaks of HE‐1,4‐DHAQ remained nearly unchanged throughout the cycling process, indicating good structural integrity and reversibility (**Figure** **4**a–c).^[^ ^41^, ^42^ ^]^ In situ UV–vis measurements revealed that the absorption peak at 286 nm (π→π* transition) showed negligible intensity changes during two full charge–discharge cycles, further confirming the outstanding structural stability of HE‐1,4‐DHAQ (Figure 4d,e; Figure S21, Supporting Information). In situ FTIR spectra provided insight into molecular bond dynamics: during discharge, the C═O stretching band at 1646 cm^−1^ significantly weakened, indicating that Zn^2+^/H^+^ insertion altered the local carbonyl environment; this band recovered upon charging, reflecting a reversible redox transition of the carbonyl group.^[^ ^43^ ^]^ Meanwhile, the O─H stretching vibration at 3319 cm^−1^ also exhibited reversible intensity changes, suggesting the reversible insertion/extraction of water molecules (Figure 4f,g). Ex situ XPS analyses further revealed the valence evolution of Mn at different voltage states (Figure 4h). As discharge progressed from point b to c, Mn^2+^ signals gradually increased while Mn^3+^ and Mn^4+^ signals decreased. From point c to e during discharge, Mn valence signals showed minimal variation. Based on CV analysis, this voltage window corresponds to the redox reactions of Cu (c → d) and O (d → e). To verify this, ex situ elemental mapping was performed, confirming the spatial and intensity changes of Cu and O signals in line with the above assignments (Figure 4i–k; Figures S22–S24, Supporting Information). During charging (f → h), this trend reversed, indicating a reversible valence transformation of Mn within HE‐1,4‐DHAQ during cycling. Additionally, as the discharge process proceeds, the Zn content in the elemental mapping gradually increases, indicating that Zn^2+^ is progressively inserted into the material structure. The Zn 2p signal in the XPS spectra becomes stronger during discharge and weakens during charge, further confirming the reversible insertion/extraction of Zn^2+^ (Figure 4l). Overall, this comprehensive suite of in situ and ex situ characterizations provides deep insight into the structural and electrochemical mechanisms of HE‐1,4‐DHAQ in AZIBs, offering valuable theoretical and experimental support for the application of HE nanomaterials in aqueous battery systems.

**Figure 5:**
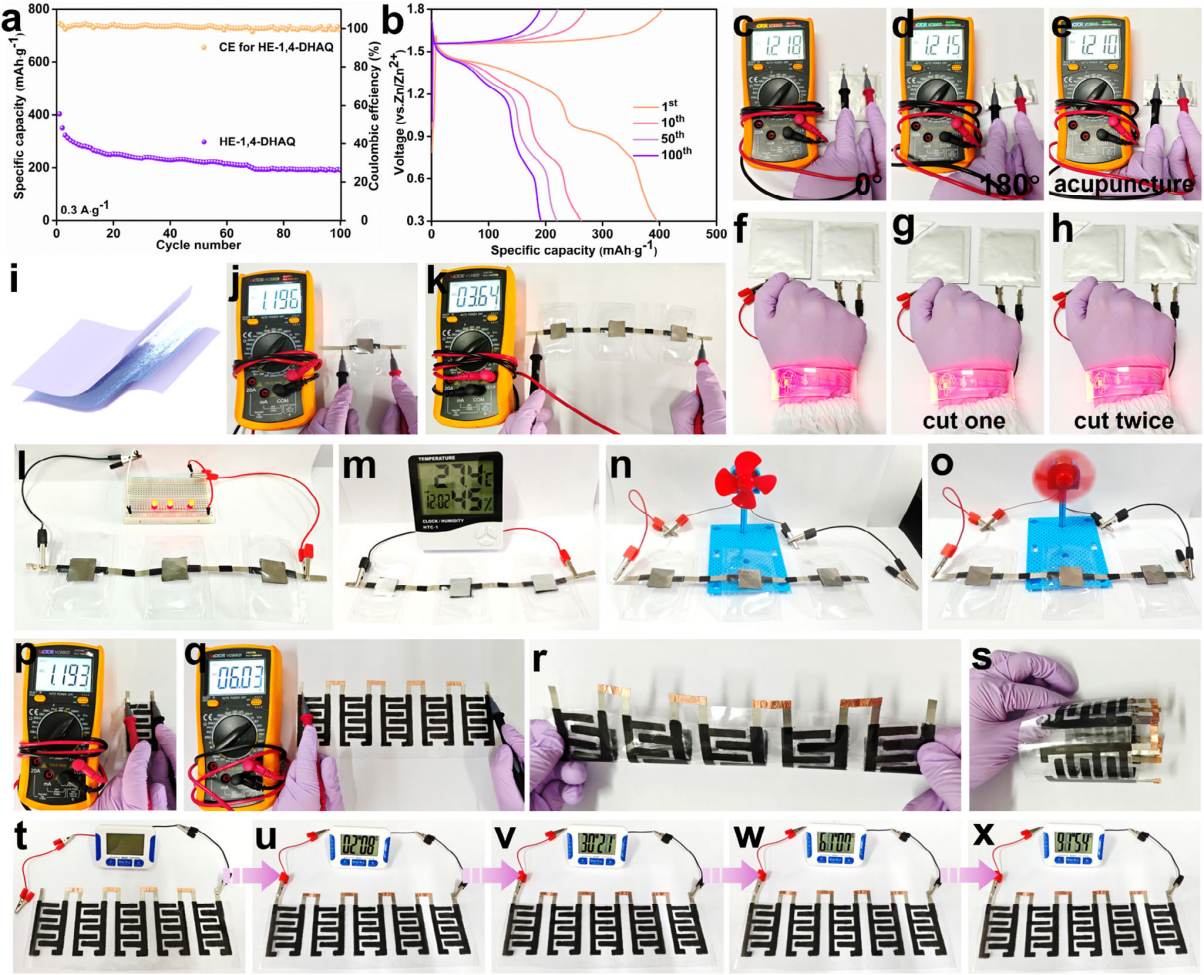
a) Cycle performance and b) charge–discharge profiles of HE‐1,4‐DHAQ soft‐pack battery. c–e) The voltage of the HE‐1,4‐DHAQ soft‐pack battery under bending at different angles and after acupuncture. f–h) The HE‐1,4‐DHAQ soft‐pack battery can power a wristband after being cut. The HE‐1,4‐DHAQ fabricated as gel electrolyte device is capable of powering l) LED light, m) hygrothermograph, and n,o) a small electric fan. The HE‐1,4‐DHAQ fabricated as a screen‐printed device can p,q) maintain a stable voltage, r,s) withstand bending at different angles, and t–x) power a timer.

The incorporation of HE nanomaterials into flexible and wearable energy storage devices remains rare in both research and practical applications. In this study, the synthesized HE‐1,4‐DHAQ is applied to flexible energy storage systems and wearable electronics, expanding its potential in aqueous battery technologies. Specifically, HE‐1,4‐DHAQ serves as the cathode material, paired with a Zn foil anode and a 2 m ZnSO_4_·7H_2_O aqueous electrolyte to assemble a soft‐pack batteries. At a current density of 0.3 A·g^−1^, the device maintains a high specific capacity of 190.1 mAh·g^−1^ after 100 cycles (**Figure** **5**a). The charge–discharge profiles closely resemble those of coin cells, confirming the excellent electrochemical performance of the soft‐pack batteries (Figure 5b). Even after undergoing 0° and 180° bending, as well as acupuncture tests, the voltage drop is negligible, indicating outstanding mechanical stability and safety (Figure 5c–e). To assess its practical applicability, two soft‐pack batteries were connected in series to power a wristband device. Remarkably, the system continued to operate stably even after one or two cutting actions, further demonstrating its high safety and reliability (Figure 5f–h). Additionally, HE‐1,4‐DHAQ was integrated with a gel electrolyte to fabricate flexible gel electrolyte devices, which delivered stable voltage outputs in both single device and three‐series device configurations (Figure 5i). Both a single device and three‐series devices maintained stable voltage (Figure 5j,k). To evaluate its practical application capabilities, the device was used to illuminate LEDs, power a hygrothermograph, as well as drive a small electric fan, demonstrating its flexibility and utility (Figure 5l–o). Furthermore, leveraging screen‐printing technology, HE‐1,4‐DHAQ was printed as a cathode on a polyethylene terephthalate (PET) substrate to fabricate a screen‐printed device. Stable power output was achieved in both single device and five‐series device configurations (Figure 5p,q). The device exhibited no electrolyte leakage or mechanical failure under various bending angles, indicating excellent mechanical robustness and safety (Figure 5r,s; Figure S25, Supporting Information). Notably, the screen‐printed devices were able to continuously power a timer for over 90 min, demonstrating exceptional practical application potential (Figure 5t–x). The PVDF binder used in this study effectively ensured the structural integrity of the electrodes during device fabrication. Future work will focus on optimizing the binder system to further enhance the stability and cycling performance of the battery.^[^ ^44^ ^]^ In summary, this work systematically verifies the outstanding electrochemical performance and mechanical stability of HE‐1,4‐DHAQ in flexible energy storage devices and provides valuable theoretical and practical insights into the application of HE nanomaterials in aqueous batteries and wearable electronics.

## 3 Conclusion

In this study, a series of nanomaterials ranging from binary to HE nanomaterials were successfully synthesized and applied to AZIBs. Among them, HE‐1,4‐DHAQ contains three synergistic active centers: ─OH groups on the ligand, Mn, and Cu. These active sites not only provide abundant electrochemical reactivity but also enhance electron and ion transport pathways through entropy stabilization and multi‐metallic cooperation. HE‐1,4‐DHAQ delivers a high specific capacity of 222.6 mAh·g^−1^ after 150 cycles at a current density of 0.3 A·g^−1^, and maintains excellent capacity retention and electrochemical stability after 3000 cycles at 1.0 A·g^−1^. By combining in situ and ex situ characterization techniques, the structural stability and electrochemical reaction mechanism of HE‐1,4‐DHAQ during charge–discharge were systematically investigated. The synergistic effect of the three active centers, along with the reversible Zn^2+^ insertion/extraction mechanism, was clearly demonstrated. Moreover, HE‐1,4‐DHAQ exhibits good safety and mechanical stability in practical applications. This work not only provides valuable insights into the application of HE materials in AZIBs but also offers a new design strategy and technical foundation for the development of next‐generation energy storage systems and wearable electronic devices.

## Conflict of Interest

The authors declare no conflict of interest.

## Supporting information



Supporting Information

## Data Availability

The data that support the findings of this study are available from the corresponding author upon reasonable request.
