# Supplementary material for: Triple Redox–Enabled High‐Entropy Metal–Organic Coordination Driving High‐Performance Aqueous Zinc–Ion Batteries
Source: Adv Sci (Weinh). 2025 Aug 12;12(42):e11748. doi: 10.1002/advs.202511748 (PMC12622408; doi:10.1002/advs.202511748)
Supplement: Supplementary file 1 — Supporting Information [file ADVS-12-e11748-s001.docx]

**Supplementary Information**

**Triple Redox–Enabled High****–Entropy Metal****–Organic Coordination Driving High–Performance Aqueous Zinc–Ion Batteries**

*Qian Li, Yanfei Zhang, Ziming Qiu, Meng Du, Shengxu Wei, Yiwen Liu, Wanchang Feng, Huan Pang****

*Q. Li, Y. Zhang, Z. Qiu, M. Du, S. Wei, Y. Liu, W. Feng, H. Pang**

School of Chemistry and Chemical Engineering, Yangzhou University, Yangzhou, 225002 Jiangsu, P. R. China.

*S. Wei*

School of Chemistry and Chemical Engineering, Chongqing University of Science and Technology, Chongqing, 401331, P. R. China

*Y. Liu*

School of Environmental Science, Nanjing Xiaozhuang University, Nanjing, 211171, Jiangsu, P. R. China.

Corresponding author.

E-mail: [panghuan@yzu.edu.cn](mailto:panghuan@yzu.edu.cn), huanpangchem@hotmail.com (H. Pang)

**General Information**

**Materials and** **general methods**

All reagents were commercially purchased and used without purification. Deionized water was used throughout the whole experiment. The X–ray diffraction (XRD) patterns was performed by Bruker AXS D8 advance with Cu Kα radiation of 40 kV (λ=1.5418 Å). Autosorb–Iq obtained the N_2_ sorption isothermals and pore size distribution *via* Brunauer–Emmet–Teller (BET) method. Scanning electron microscopy (SEM) images were obtained by Zeiss-Supra 55 microscopes at an acceleration voltage of 5 kV. JEM–2100 microscope obtained transmission electron microscopy (TEM) images with an acceleration voltage of 200 kV. Energy dispersive X–ray (EDX), and elemental mapping were captured on a Tecnai G2 F30 at an acceleration voltage of 300 kV. X–ray photoelectron spectroscopy (XPS) analysis was carried out using a Thermo Scientific ESCALAB 250Xi X–ray photoelectron spectrometer with Al Kα radiation of 1486.6 eV as the excitation source, and the survey thickness is 2-3 nm. The reference for calibration is the peak of C 1s at 284.8 eV. Thermogravimetric analysis (TGA) was performed on a TGA Q50 instrument with a heating rate of 5 °C min^–1^ in the N_2_ atmosphere. Fourier transform infrared (FTIR) spectra were obtained on a BRUKER-EQUINOX-55 IR spectrophotometer. Inductively Coupled Plasma-Optical Emission Spectrometry (ICP–OES) analysis was performed by Optima 7300 DV. Ultraviolet-visible spectrophotometer (UV–Vis) diffuse reflectance spectra was carried out as a UV–2550 (Shimadzu) spectrometer.

**Electrochemical test**

The electrochemical performances were measured in coin cells. The working electrodes were prepared by mixing polyvinylidene fluoride (PVDF, 10 wt.%), Super P carbon (20 wt.%) and active materials (70 wt.%) in N-methyl-2 pyrrolidone (NMP) solvent on titanium foil which was used as the current collectors. The coated electrode was dried in vacuum at 60 ℃ for 12 h. The separator was glass fiber. 2 M ZnSO_4_∙7H_2_O aqueous solution as the electrolyte. Statistically, the load quality of the active material is 0.7 mg, the thickness of the zinc foil is 0.1 mm, and the electrode is circular with a diameter of 12 mm. The cycling performance and rate capability were tested by a battery measurement system (CT2001A, Wuhan Land, China) at room temperature. The cyclic voltammetry (CV) and electrochemical impedance spectroscopy (EIS) were tested on a CHI 760D electrochemical workstation (Shanghai Chenhua), and the CV curves was obtained in the potential range from 0.8 to 1.8 V. Before CV curves testing, activate with low current density. The EIS measurements were performed at a frequency ranging from 0.01 Hz to 100 kHz with an amplitude of 5 mV. Open circuit potential was read and used. In the amount of cathode material HE-1,4-DHAQ was approximately 10 mg for each soft−pack battery, about 2.5 mg for each gel electrolyte device, and around 9 mg for each screen−printed device.

**Synthesis of materials**

**Synthesis of MnCo-1,4DHAQ**

Dissolve 0.275 mmol of 1,4-DHAQ in a mixed solvent of 24 mL DMF and 3 mL ethanol and stir for 20 min to ensure complete dissolution. Subsequently, add Mn(CH_3_COO)_2_∙4H_2_O and Co(CH_3_COO)_2_∙4H_2_O to the solution in a molar ratio of 2 mmol:1 mmol, and continue stirring for 30 min to achieve homogeneous mixing. Transfer the resulting mixture into a Teflon–lined autoclave and react at 180 ℃ for 12 h. After the reaction, allow the autoclave to cool naturally to room temperature, and wash the product several times with ethanol and DMF. Finally, dry the product in an oven at 60 ℃ to obtain the target MnCo-1,4-DHAQ.

**Synthesis of MnCoNi-1,4DHAQ**

Dissolve 0.275 mmol of 1,4-Dihydroxyanthraquinone (1,4-DHAQ) in a mixed solvent of 24 mL DMF and 3 mL ethanol and stir for 20 min to ensure complete dissolution. Subsequently, add Mn(CH_3_COO)_2_∙4H_2_O, Co(CH_3_COO)_2_∙4H_2_O and Ni(CH_3_COO)_2_∙4H_2_O to the solution in a molar ratio of 2 mmol:1 mmol:1 mmol, and continue stirring for 30 min to achieve homogeneous mixing. Transfer the resulting mixture into a Teflon–lined autoclave and react at 180 ℃ for 12 h. After the reaction, allow the autoclave to cool naturally to room temperature, and wash the product several times with ethanol and DMF. Finally, dry the product in an oven at 60 ℃ to obtain the target MnCoNi-1,4-DHAQ.

**Synthesis of Mn****CoNiFe-1,4DHAQ**

Dissolve 0.275 mmol of 1,4-Dihydroxyanthraquinone (1,4-DHAQ) in a mixed solvent of 24 mL DMF and 3 mL ethanol and stir for 20 min to ensure complete dissolution. Subsequently, add Mn(CH_3_COO)_2_∙4H_2_O, Co(CH_3_COO)_2_∙4H_2_O, Ni(CH_3_COO)_2_∙4H_2_O and Fe(CH_3_COO)_3_∙nH_2_O to the solution in a molar ratio of 2 mmol:1 mmol:1 mmol:1 mmol, and continue stirring for 30 min to achieve homogeneous mixing. Transfer the resulting mixture into a Teflon–lined autoclave and react at 180 ℃ for 12 h. After the reaction, allow the autoclave to cool naturally to room temperature, and wash the product several times with ethanol and DMF. Finally, dry the product in an oven at 60 ℃ to obtain the target MnCoNiFe-1,4-DHAQ.

**Synthesis of HE-1,4DHAQ**

Dissolve 0.275 mmol of 1,4-Dihydroxyanthraquinone (1,4-DHAQ) in a mixed solvent of 24 mL DMF and 3 mL ethanol and stir for 20 min to ensure complete dissolution. Subsequently, add Mn(CH_3_COO)_2_∙4H_2_O, Co(CH_3_COO)_2_∙4H_2_O, Ni(CH_3_COO)_2_∙4H_2_O, Fe(CH_3_COO)_3_∙nH_2_O, and Cu(NO_3_)_2_∙3H_2_O to the solution in a molar ratio of 2 mmol:1 mmol:1 mmol:1 mmol:1 mmol, and continue stirring for 30 min to achieve homogeneous mixing. Transfer the resulting mixture into a Teflon–lined autoclave and react at 180 ℃ for 12 h. After the reaction, allow the autoclave to cool naturally to room temperature, and wash the product several times with ethanol and DMF. Finally, dry the product in an oven at 60 ℃ to obtain the targetHE-1,4-DHAQ.


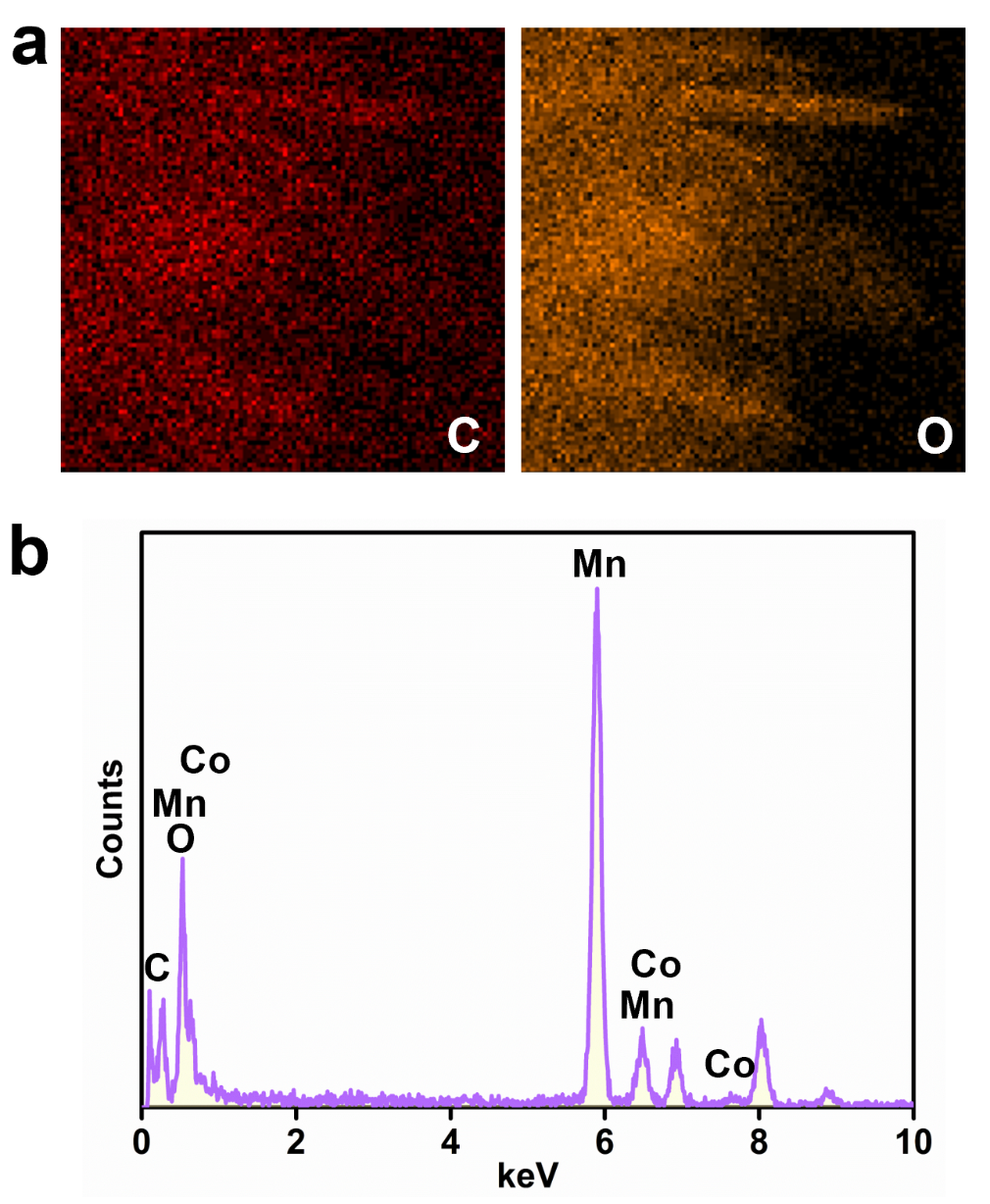


**Figures S1.** (a) Elemental mapping images and (b) EDX image of the MnCo-1,4-DHAQ.


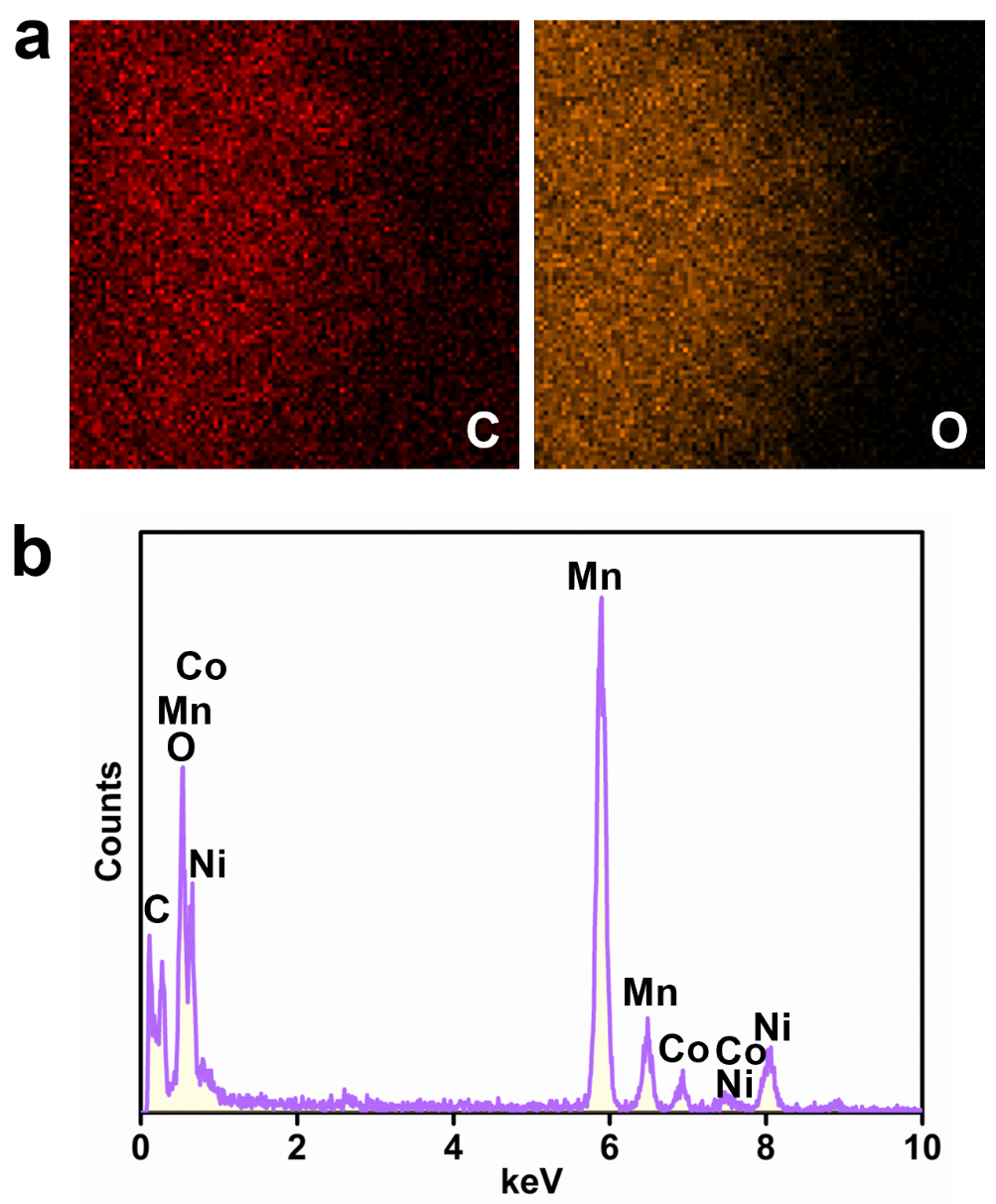


**Figures S2.** (a) Elemental mapping images and (b) EDX image of the MnCoNi-1,4-DHAQ.


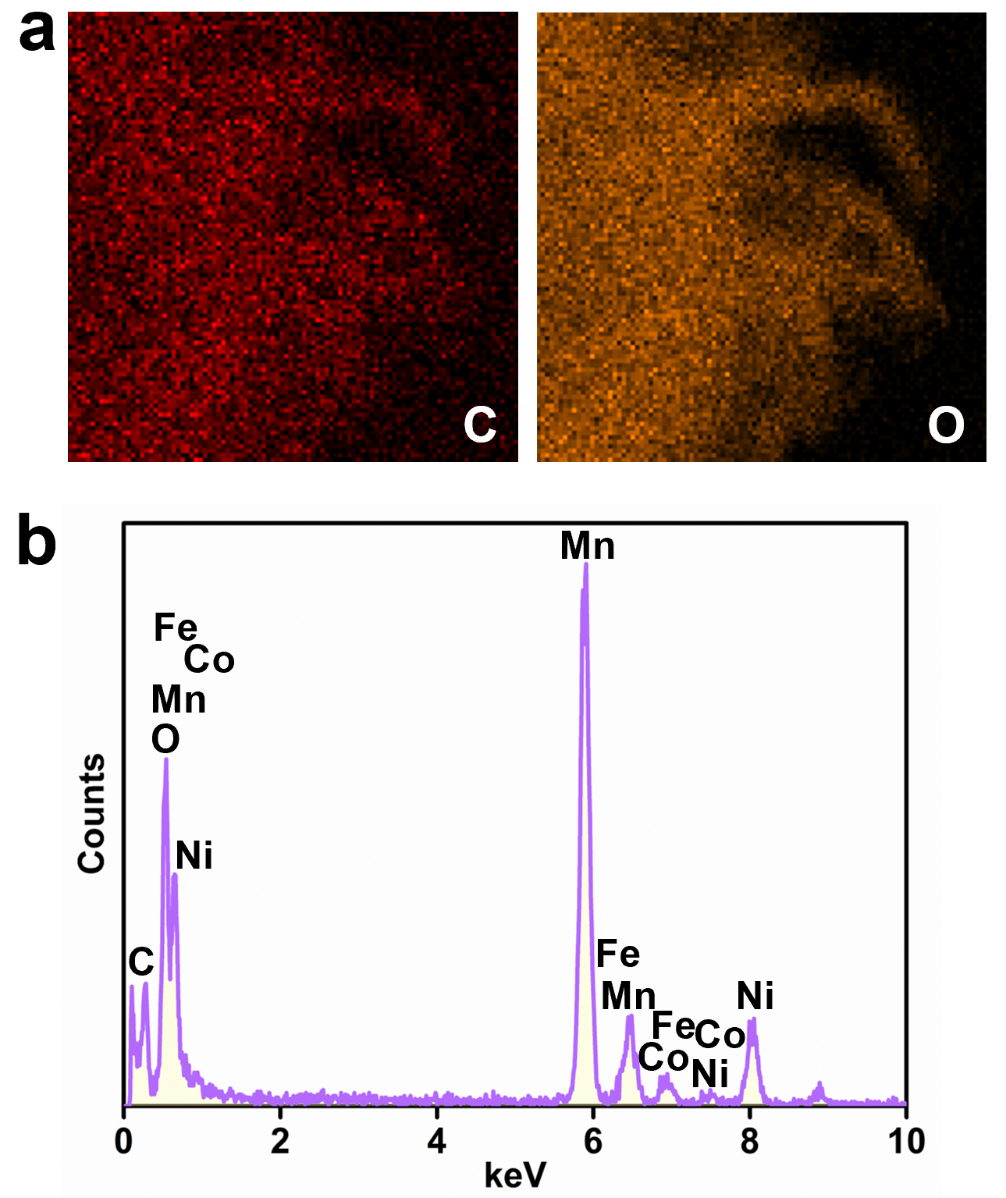


**Figures S3.** (a) Elemental mapping images and (b) EDX image of the MnCoNiFe-1,4-DHAQ.


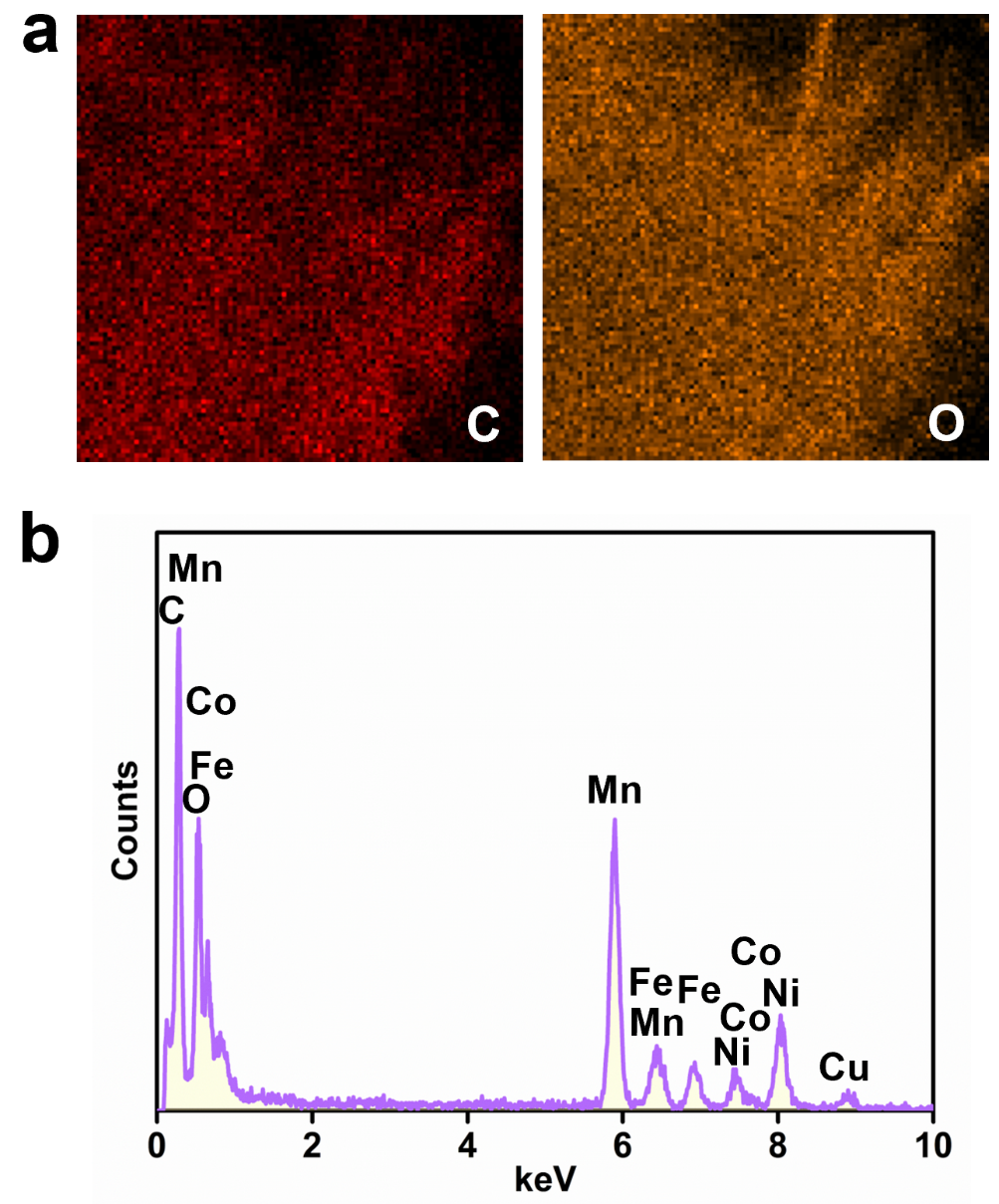


**Figures S4.** (a) Elemental mapping images and (b) EDX image of the HE-1,4-DHAQ.


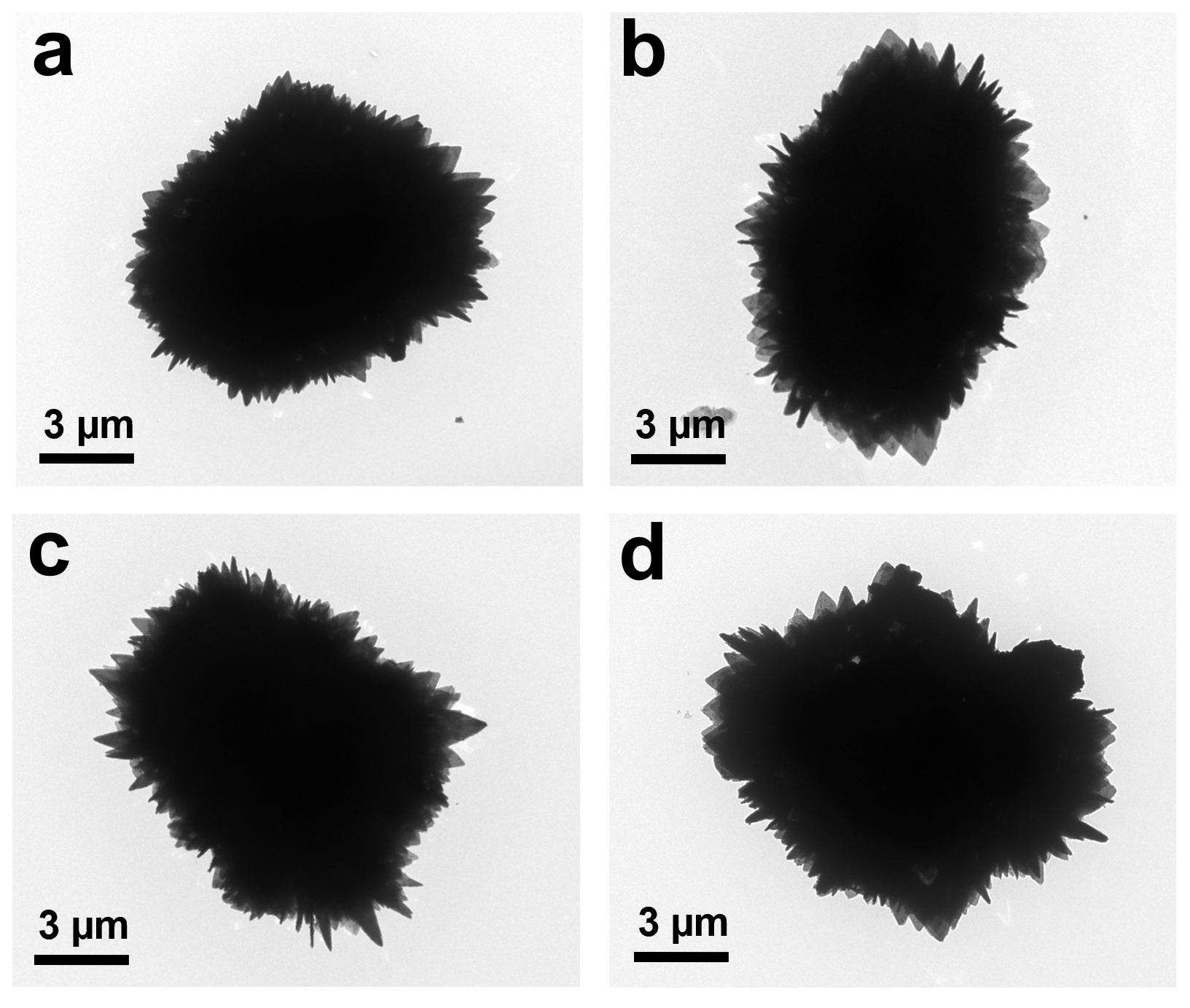


**Figures S5.** TEM images of (a) MnCo-1,4-DHAQ, (b) MnCoNi-1,4-DHAQ, (c) MnCoNiFe-1,4-DHAQ, and (d) HE-1,4-DHAQ.


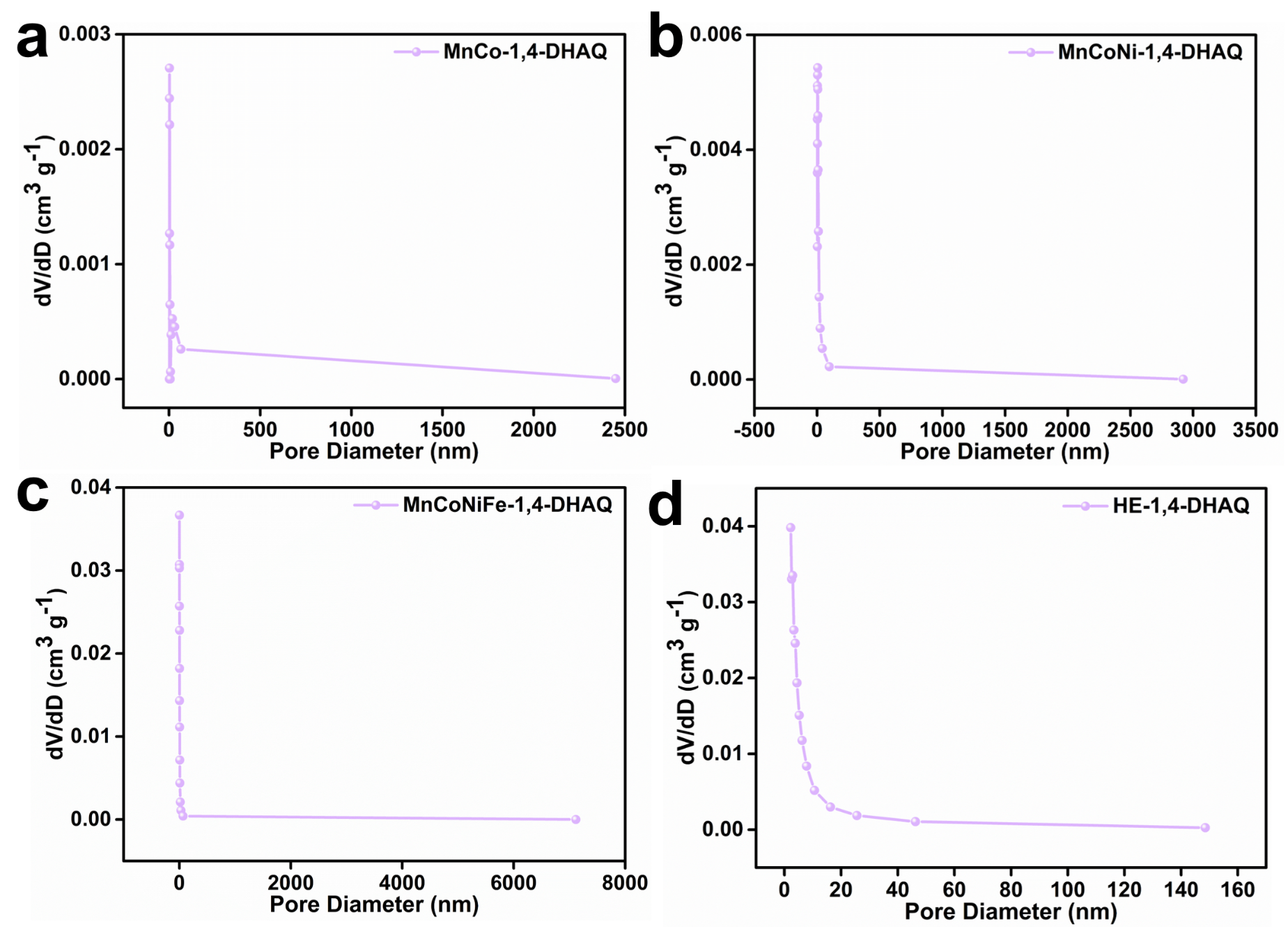


**Figures S6.** Pore size characteristics of (a) MnCo-1,4-DHAQ, (b) MnCoNi-1,4-DHAQ, (c) MnCoNiFe-1,4-DHAQ, and (d) HE-1,4-DHAQ.


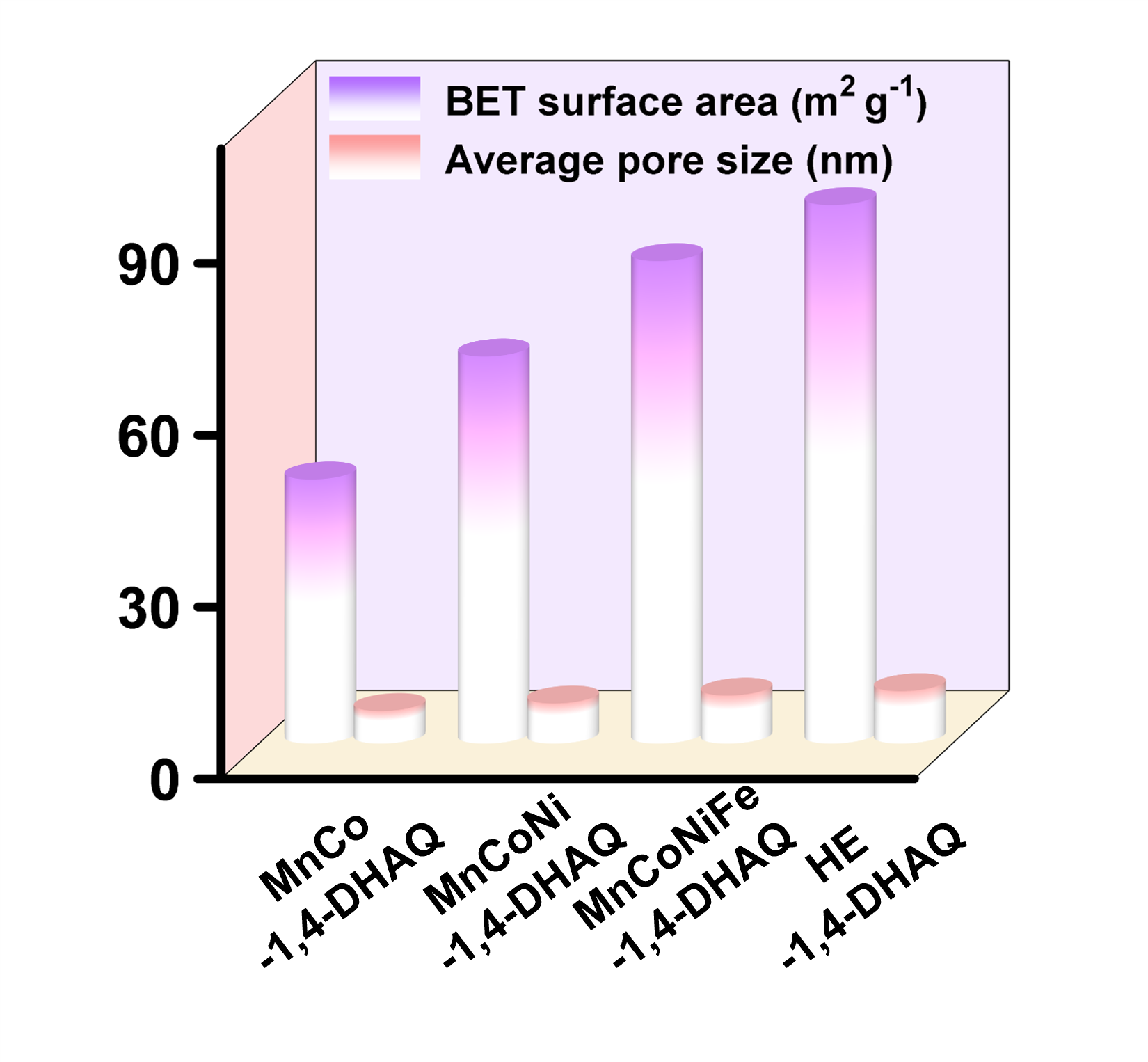


**Figure S7.** Bar chart of N_2_ adsorption–desorption isotherms and pore size characteristics of synthetic materials.

**Table S1**. The BET surface area and pore volume of prepared samples.


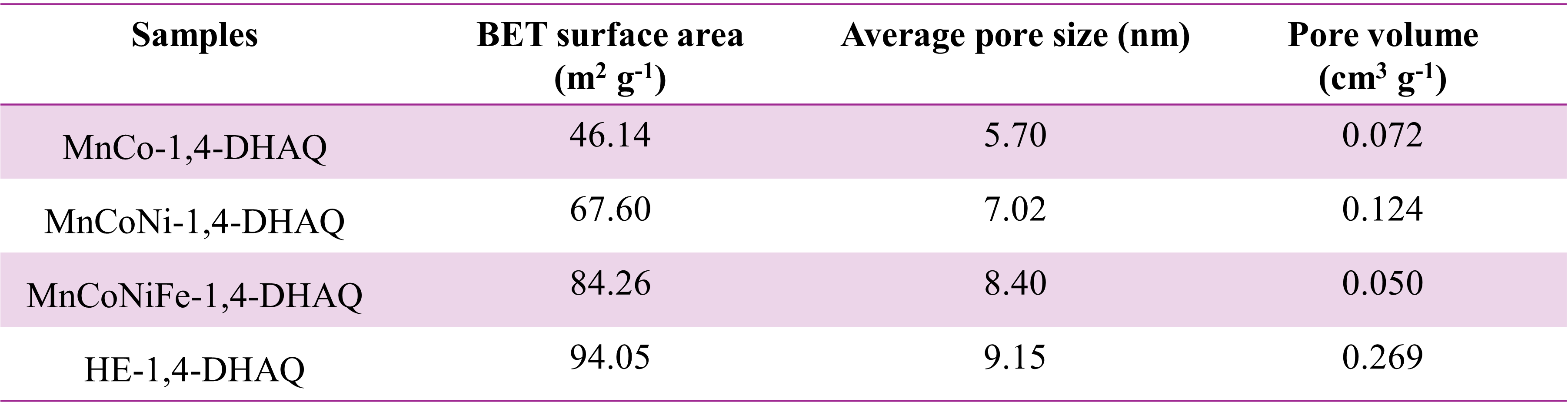


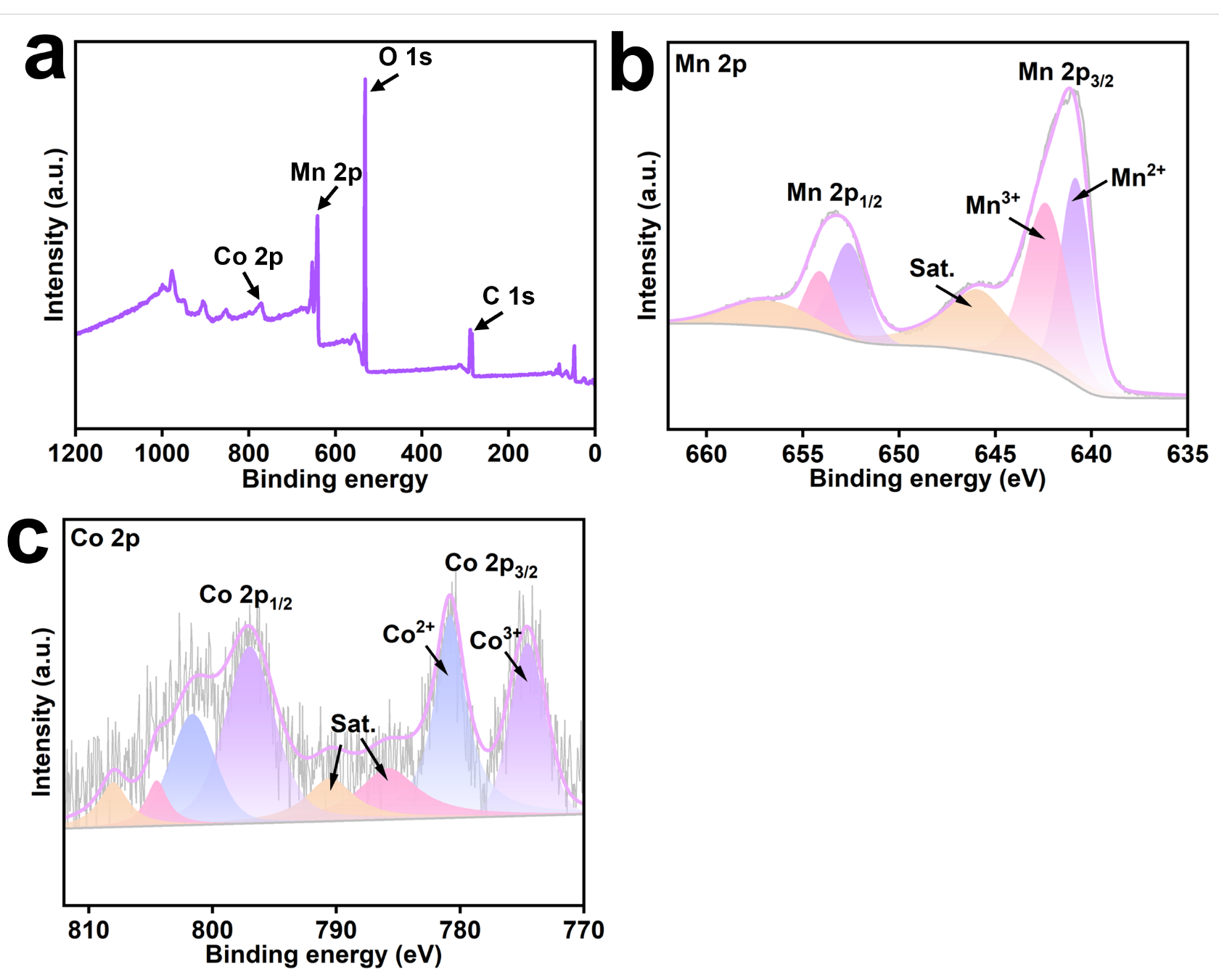


**Figures S8.** XPS spectra of MnCo-1,4-DHAQ: (a) the full-scan XPS spectrum, (b) Mn 2p, and (c) Co 2p.


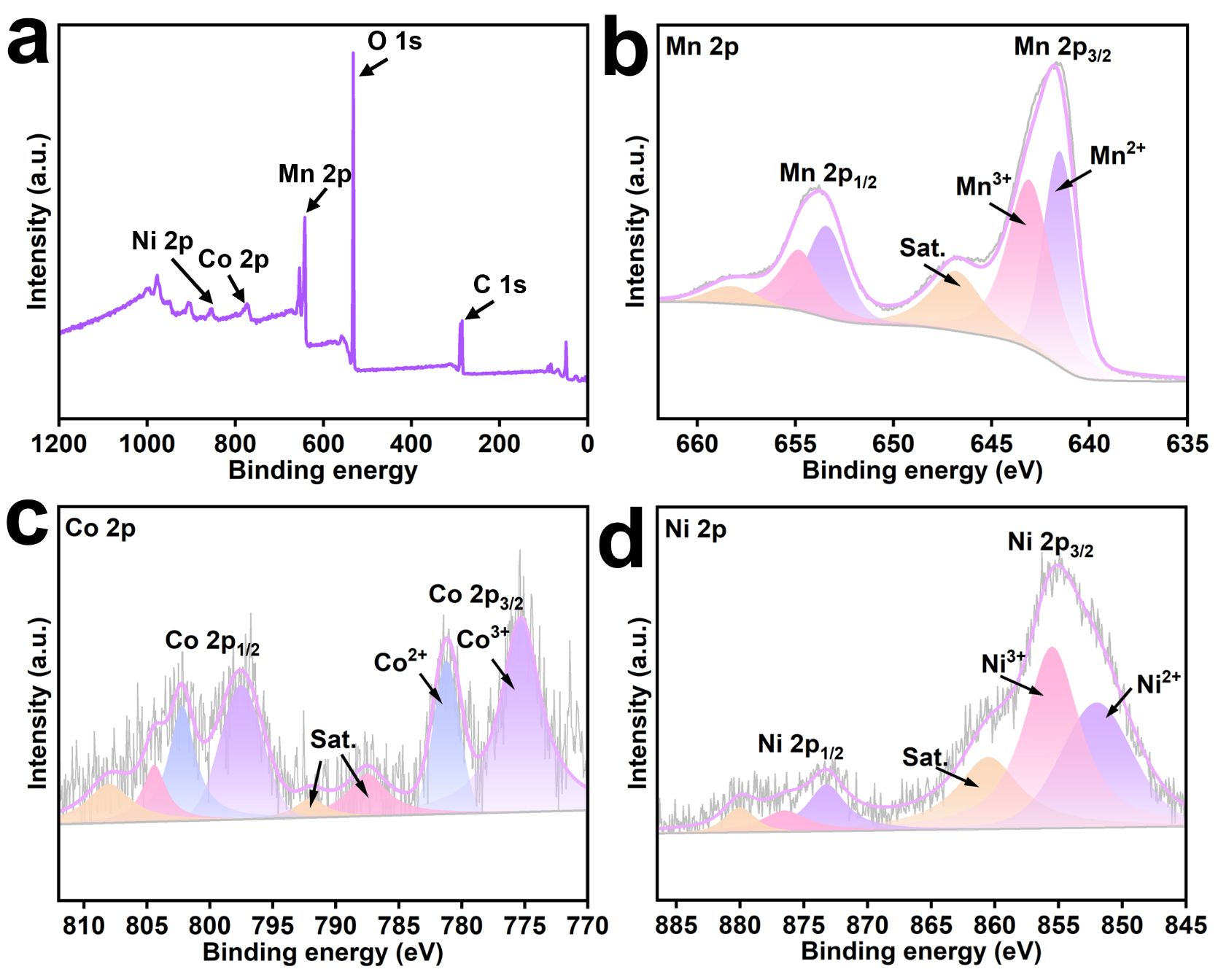


**Figures S9.** XPS spectra of MnCoNi-1,4-DHAQ: (a) the full-scan XPS spectrum, (b) Mn 2p, (c) Co 2p, and (d) Ni 2p.


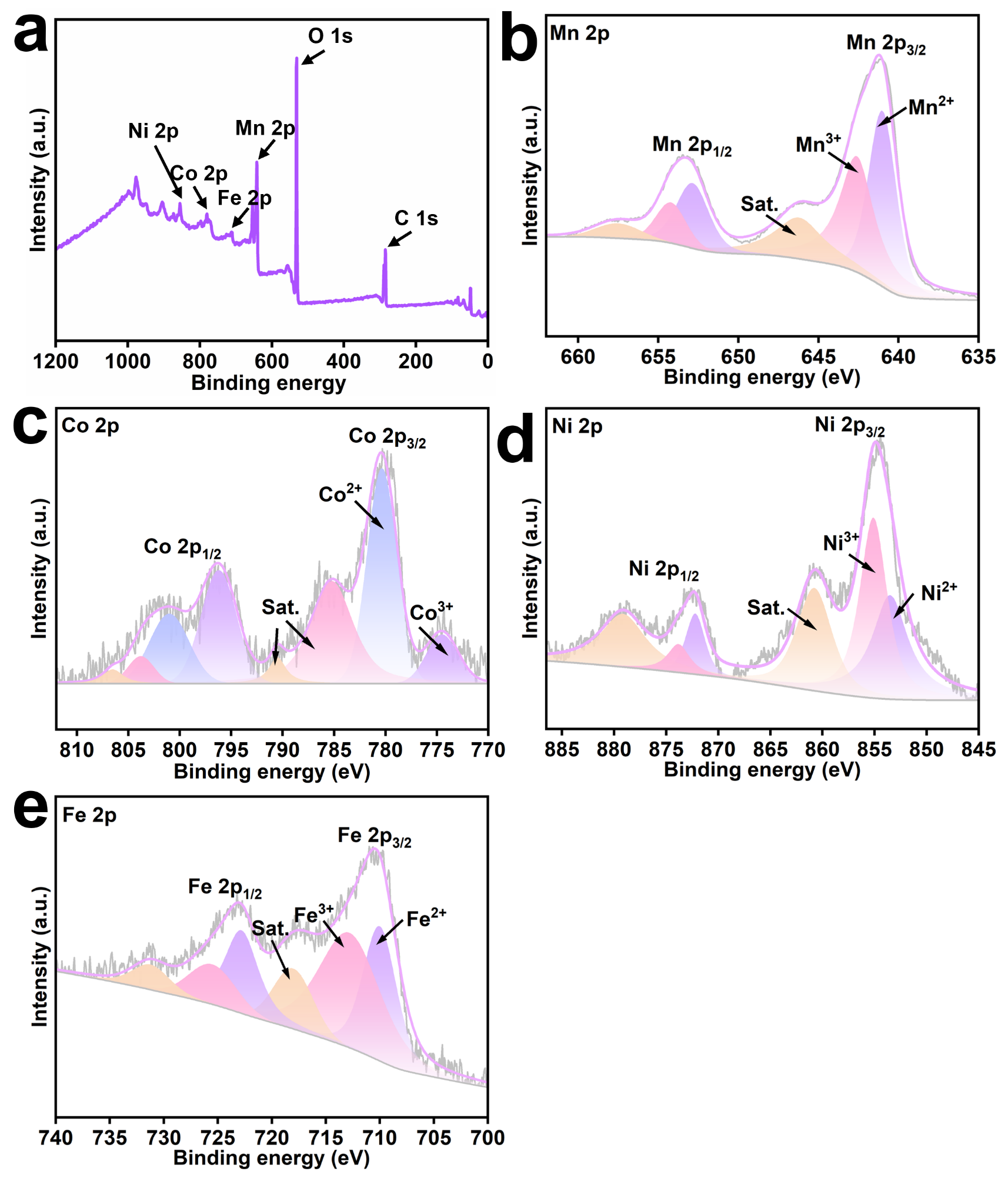


**Figures S10.** XPS spectra of MnCoNiFe-1,4-DHAQ: (a) the full-scan XPS spectrum, (b) Mn 2p, (c) Co 2p, (d) Ni 2p, and (e) Fe 2p.


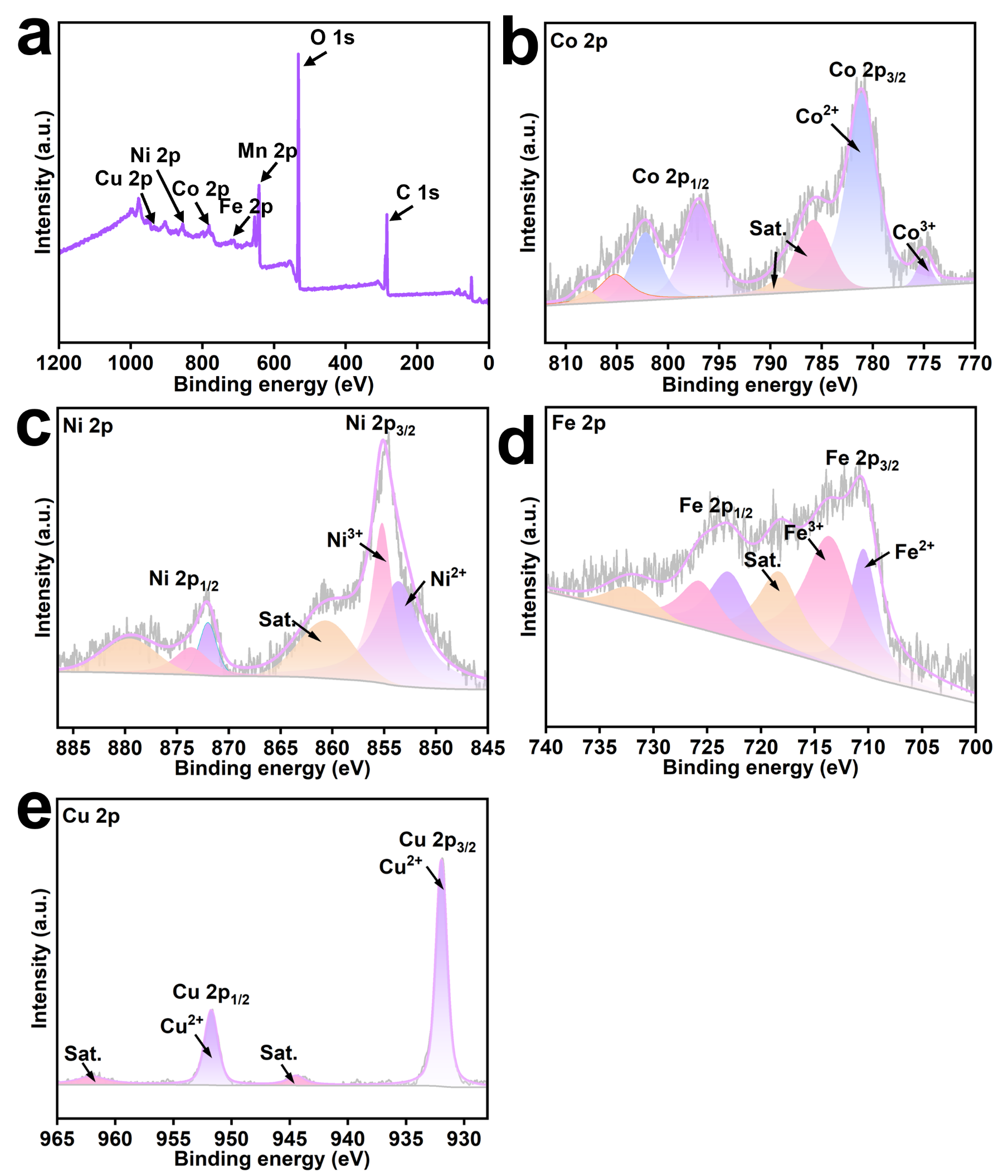


**Figures S11.** XPS spectra of HE-1,4-DHAQ: (a) the full-scan XPS spectrum, (b) Co 2p, (c)Ni 2p, (d) Fe 2p, and (e) Cu 2p.

**Table S2**. ICP-OES Analyses of synthetic materials.


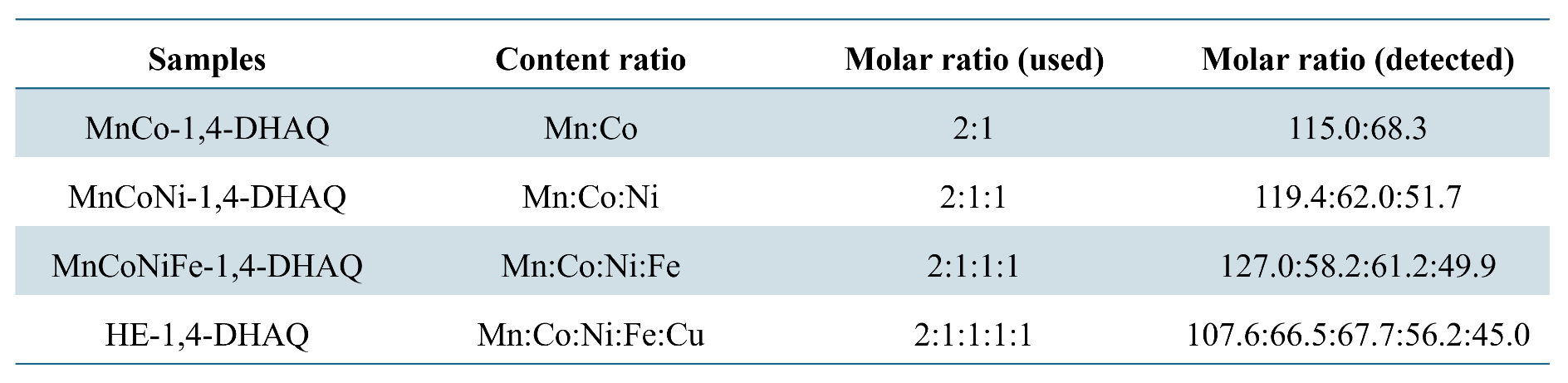


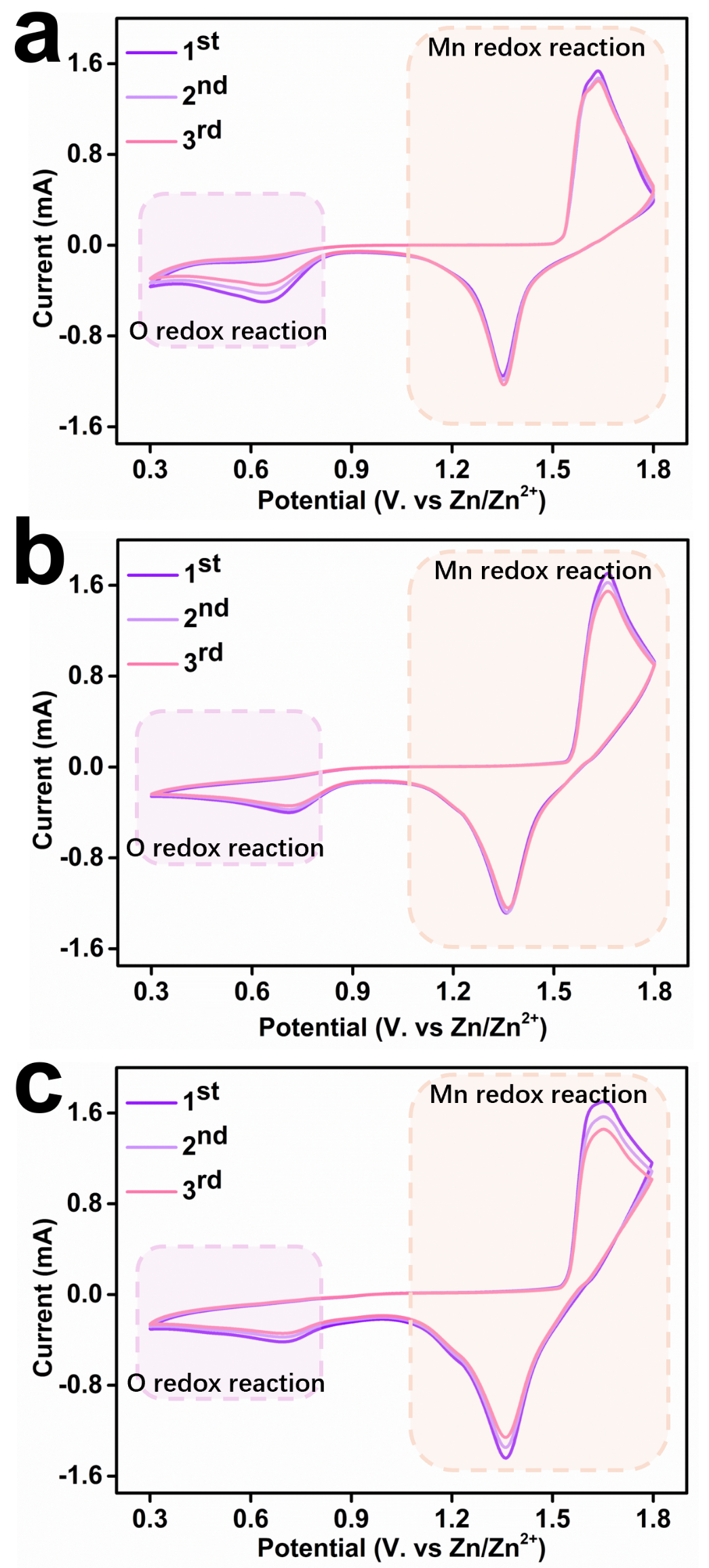


**Figures S12.** CV curves at a scan of 0.6 mV·s^–1^ of (a) MnCo-1,4-DHAQ, (b) MnCoNi-1,4-DHAQ, and (c) MnCoNiFe-1,4-DHAQ.


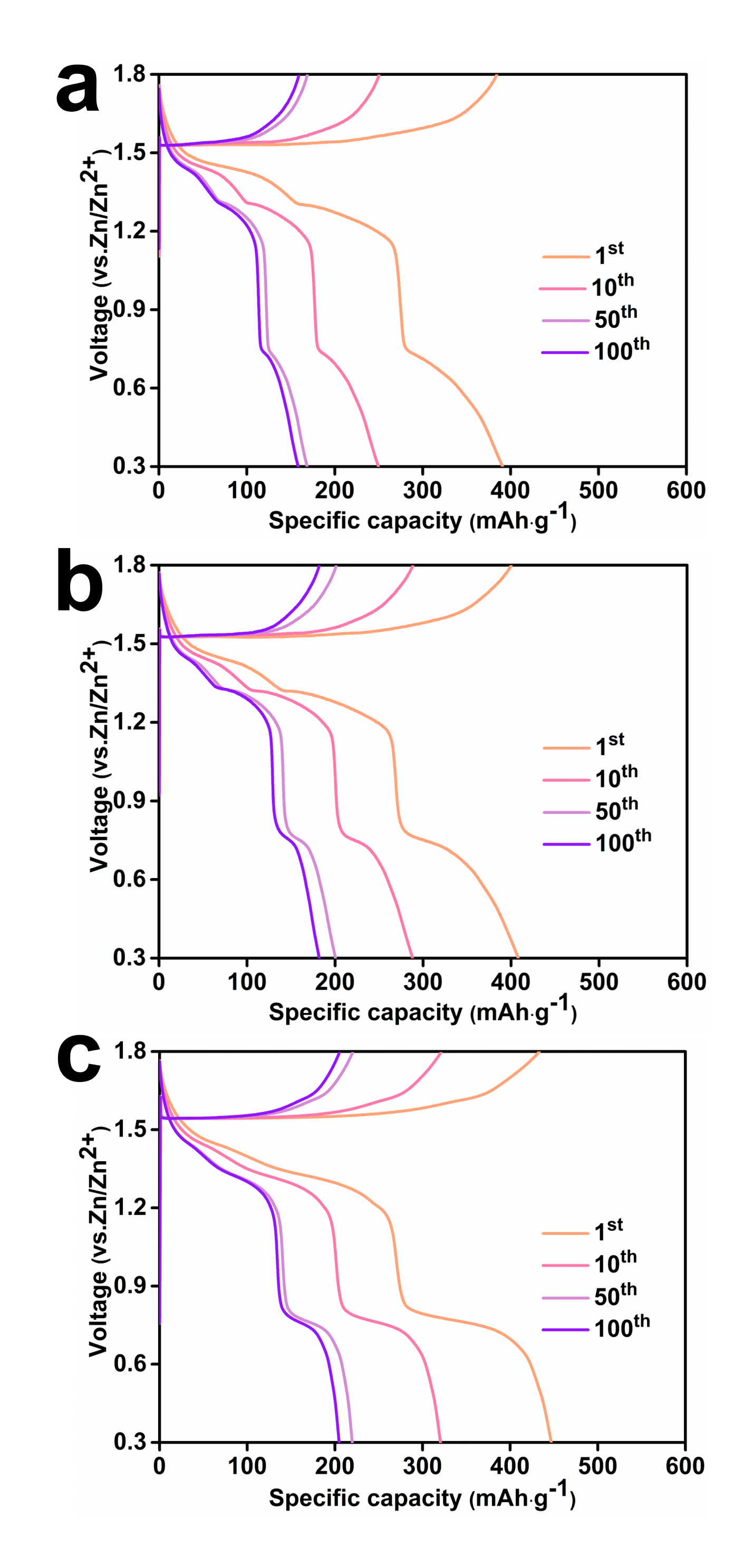


**Figures S13.** Charge–discharge profiles for the selected cycle of (a) MnCo-1,4-DHAQ, (b) MnCoNi-1,4-DHAQ, and (c) MnCoNiFe-1,4-DHAQ between 0.3–1.8 V *vs.*Zn/Zn^2+^ at the current density of 0.3 A·g^–1^.





**Figure S14.** Charge–discharge profiles of HE-1,4-DHAQ at the 150th cycle under a current density of 0.3 A·g^–1^ in electrolytes of varying concentrations.





**Figure S15.** Radar map of the capacities of synthetic materials at 0.3 A·g^–1^.





**Figure S16.** Cycling performance of Mn-1,4-DHAQ at 0.3 A·g^–1^.


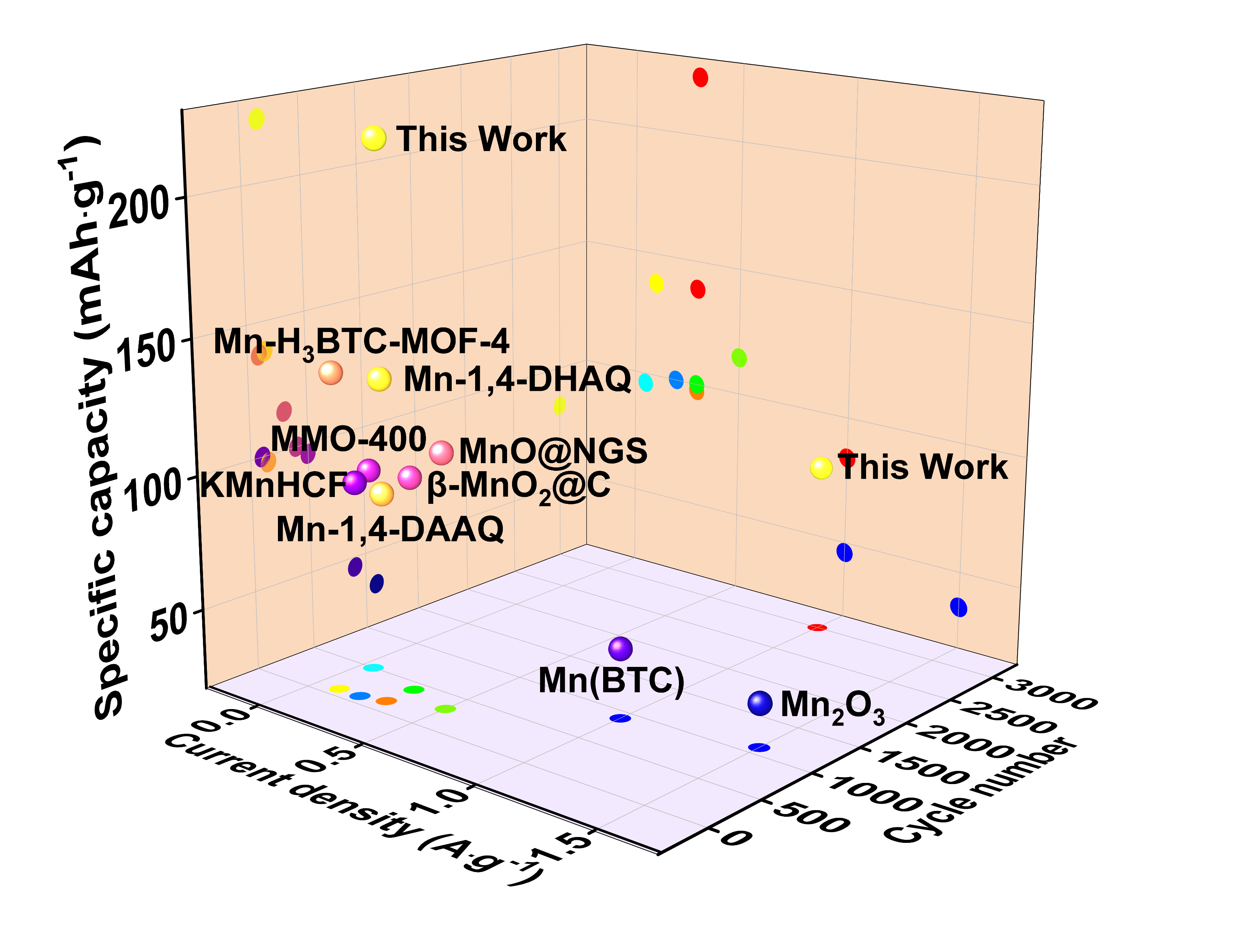


**Figure S17.** Ragone plots of HE-1,4-DHAQ and another cathode for AZIBs. References are 1-8.


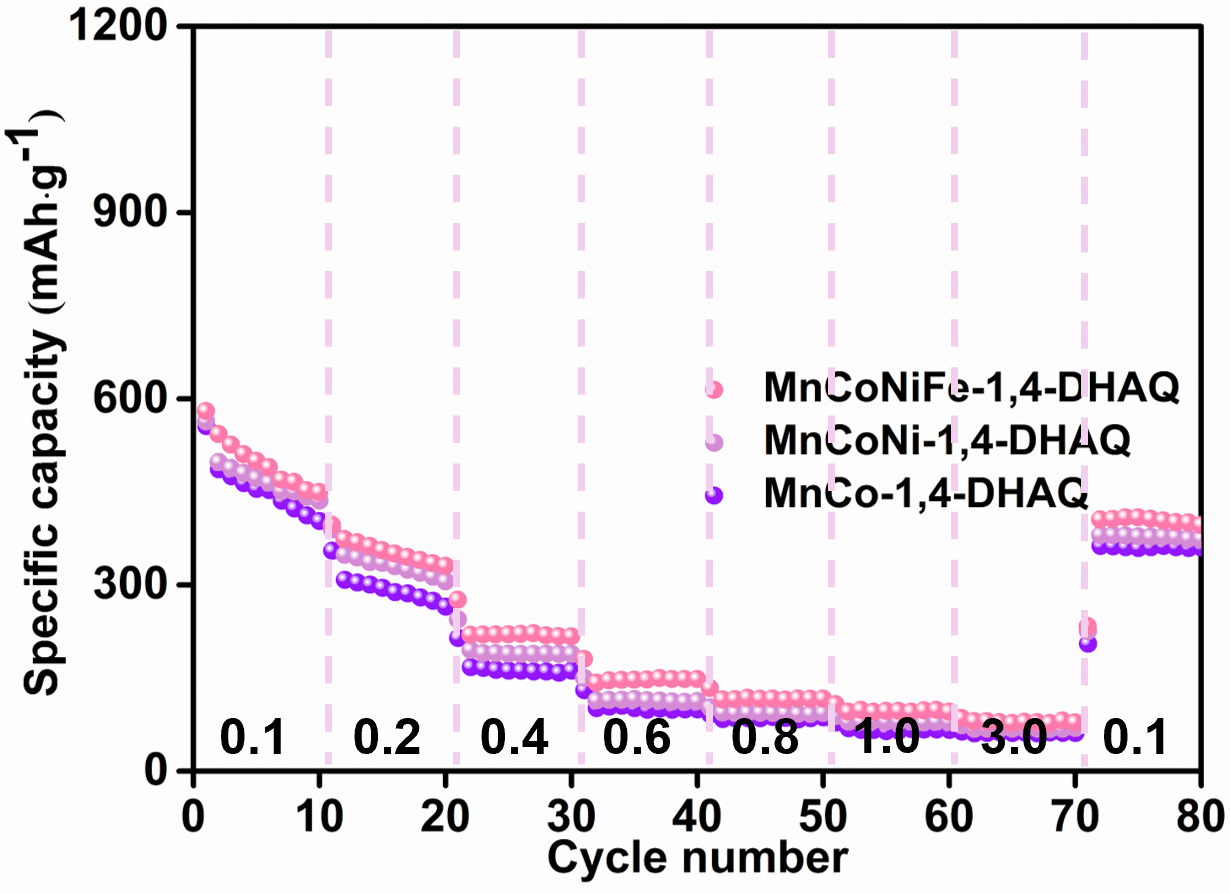


**Figure S18.** Rate performance of synthetic materials.





**Figure S19.** Cycling performance of synthetic materials at 1.0 A·g^–1^.


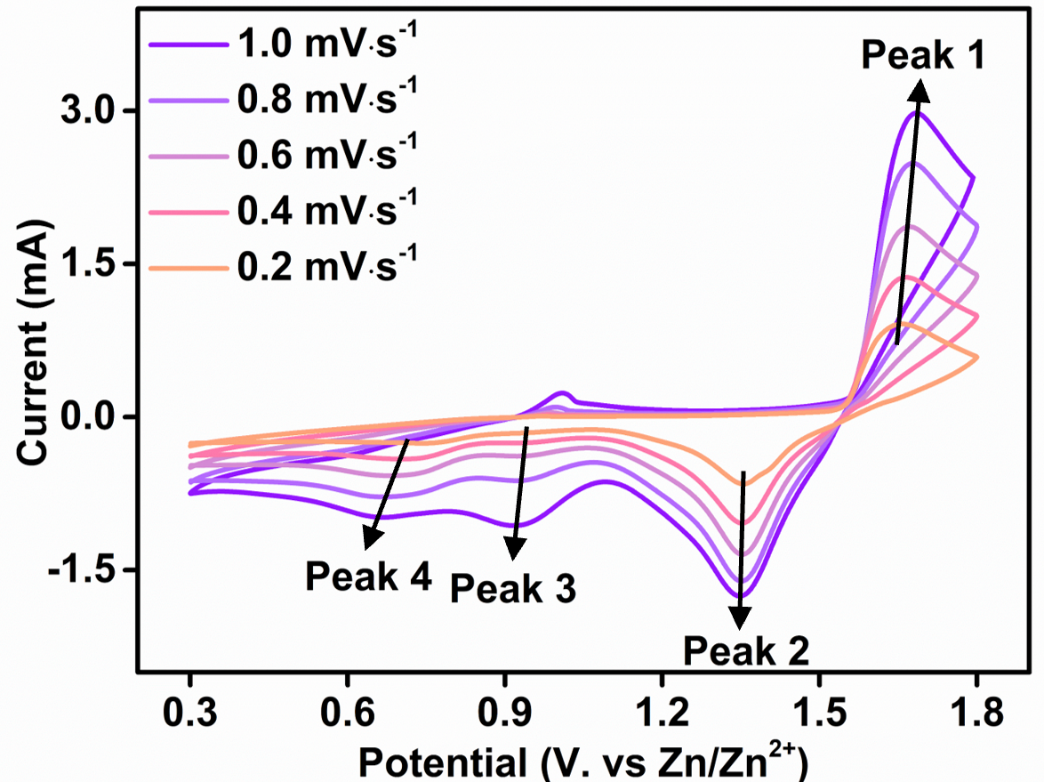


**Figure S20.** Contour plots of CV patterns of HE-1,4-DHAQ.


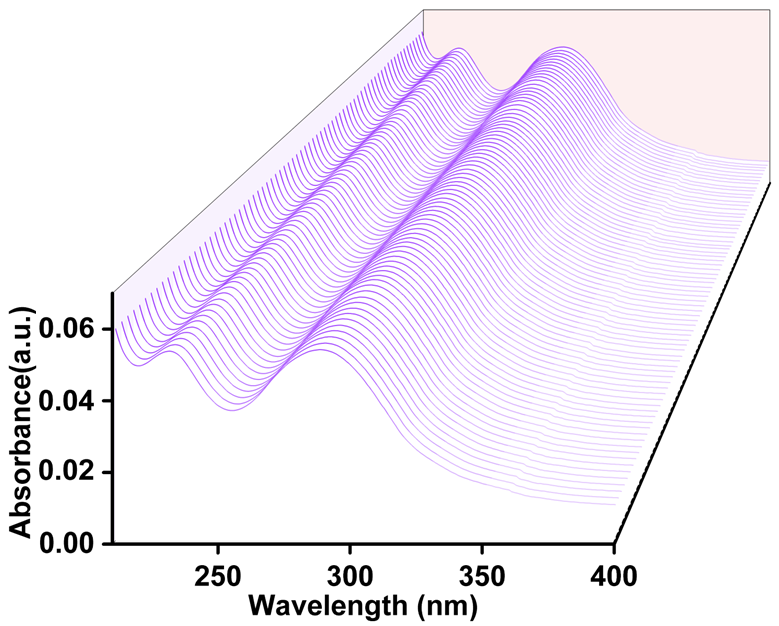


**Figure S21.** In situ UV-vis spectroscopy of HE-1,4-DHAQ during charge-discharge cycling.


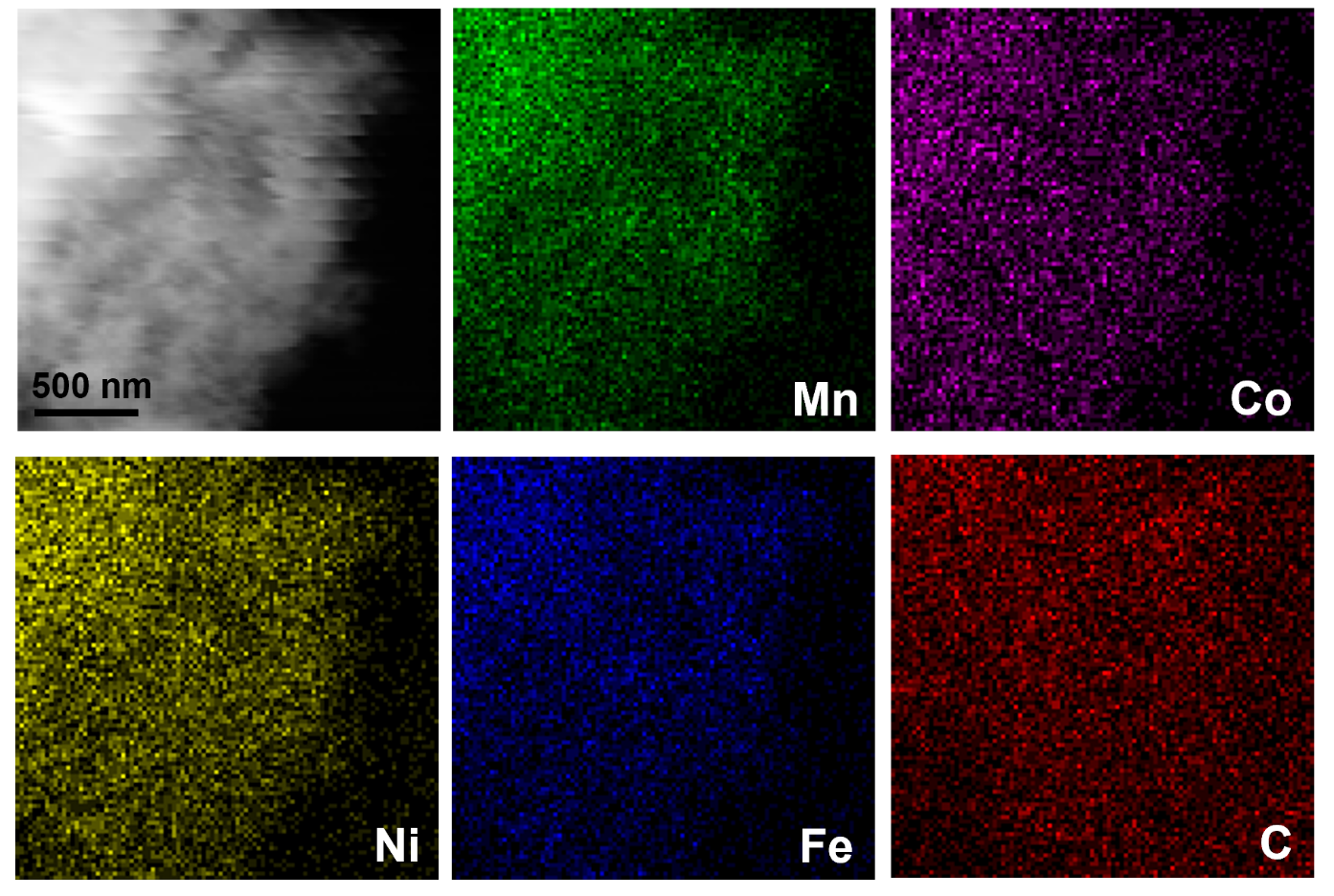


**Figure S22.** Elemental mapping images of HE-1,4-DHAQ at discharge to 1.48 V.


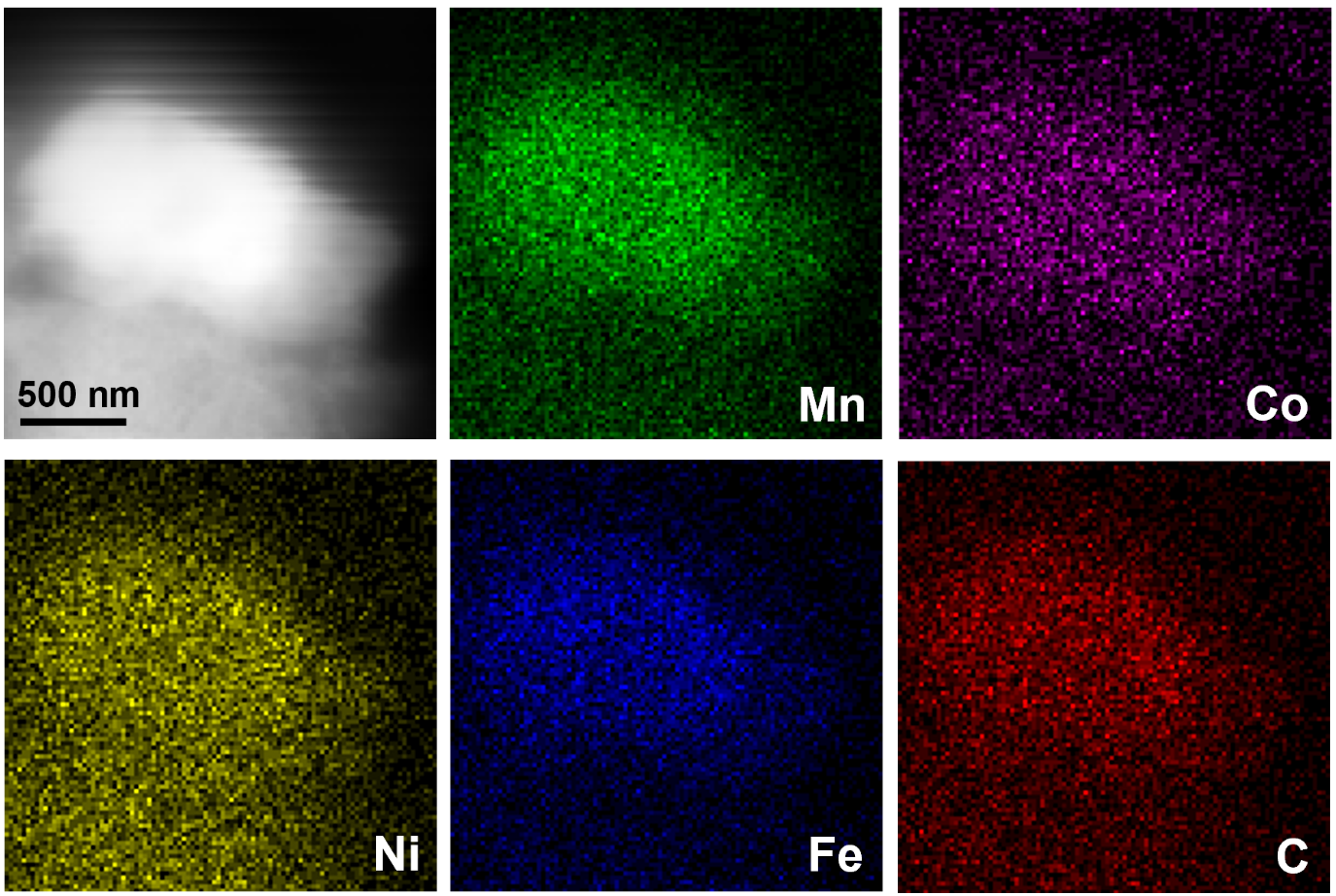


**Figure S23.** Elemental mapping images of HE-1,4-DHAQ at discharge to 1.1 V.


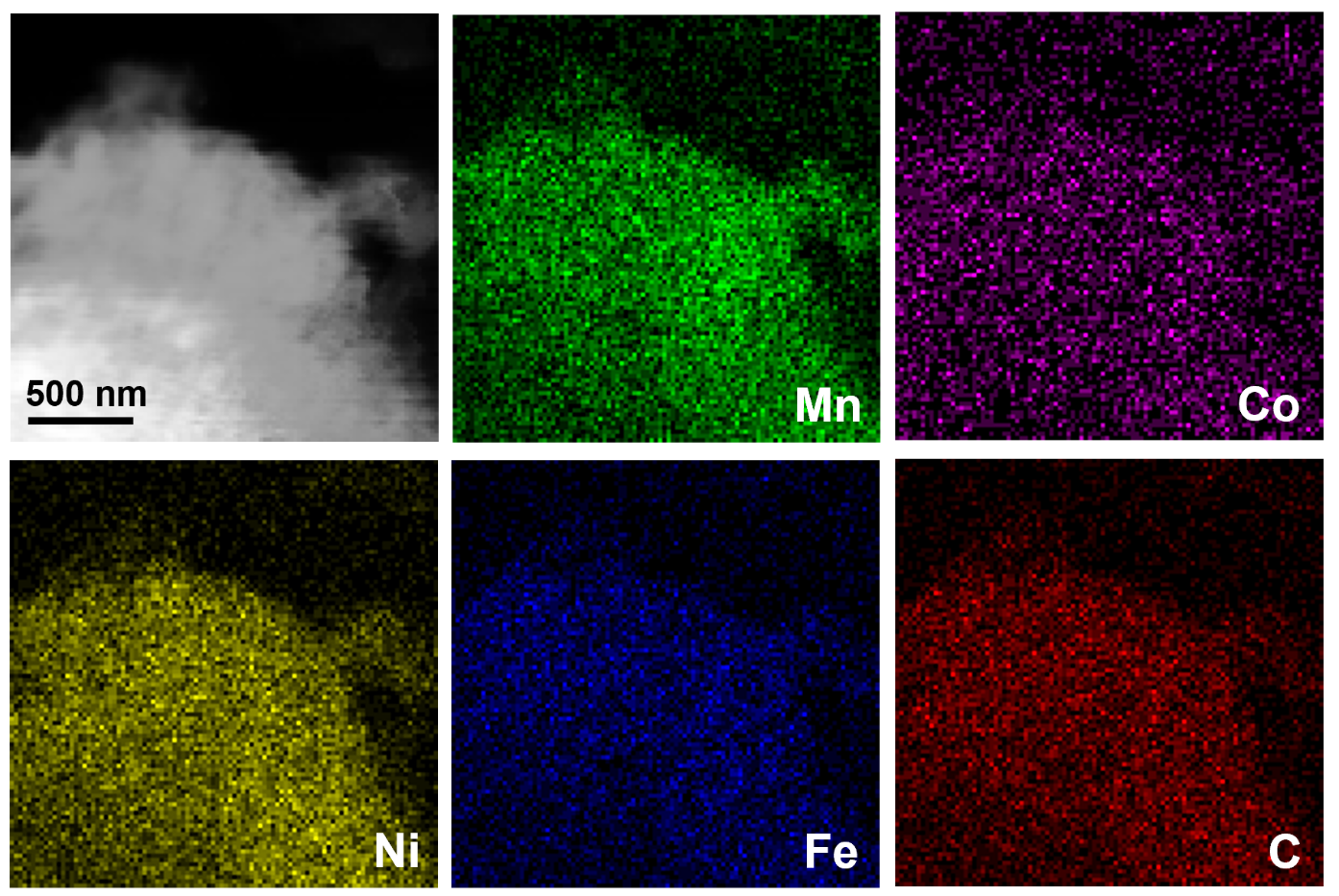


**Figure S24.** Elemental mapping images of HE-1,4-DHAQ at discharge to 0.82 V.


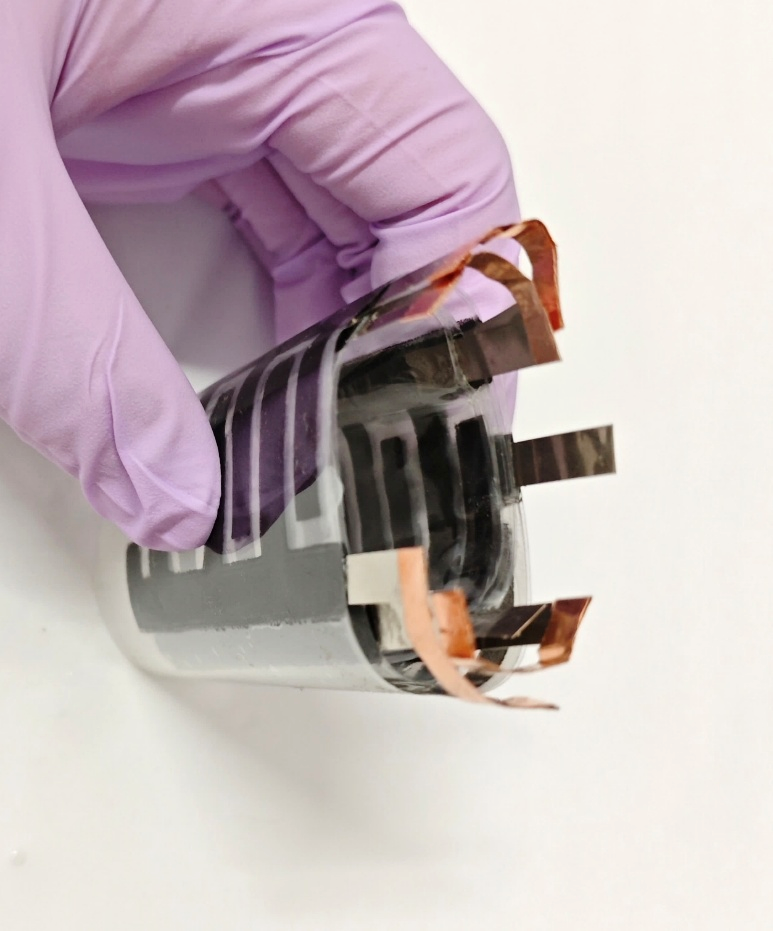


**Figure S25.** The digital photograph of five internally connected miniature batteries was obtained using screen-printing.

**References**

[1] Q. Li, Y. F. Zhang, W. C. Feng, J. F. Huang, S. X. Wei, G. Chen, Y. W. Liu, M. Du, C. H. Yin, Z. B. Yang, Y. Y. Sun, S. Cao, C. G. Pei, H. C. Chen, H. Pang, *Adv. Mater.* **2025**, 2507951.

[2] C. J. Yin, C. L. Pan, X. B. Liao, Y. S. Pan, L. Yuan, *ACS Appl. Mater. Interfaces* **2021**, *13*, 35837.

[3] W. J. Li, X. Gao, Z. Y. Chen, R. T. Guo, G. Q. Zou, H. S. Hou, W. T. Deng, X. B. Ji, J. Zhao, *Chem. Eng. J.* **2020**, *402*, 125509.

[4] W. Jiang, X. Xu, Y. Liu, L. Tan, F. Zhou, Z. Xu, R. Hu, *J. Alloy. Compd.* **2020**, *827*, 154273.

[5] V. Soundharrajan, B. Sambandam, S. Kim, V. Mathew, J. Jo, S. Kim, J. Lee, S. Islam, K. Kim, Y. K. Sun, J. Kim, *ACS Energy Lett.* **2018**, *3*, 1998.

[6] W. J. Deng, Z. G. Li, Y. K. Ye, Z. Q. Zhou, Y. B. Li, M. Zhang, X. R. Yuan, J. Hu, W. G. Zhao, Z. Y. Huang, C. Li, H. B. Chen, J. X. Zheng, R. Li, *Adv. Energy Mater.* **2020**, *11*, 2003639.

[7] X. Pu, B. Jiang, X. Wang, W. Liu, L. Dong, F. Kang, C. Xu, *Nano-Micro Lett*. **2020**, *12*, 152.

[8] L. Gou, K. Mou, X. Fan, M. Zhao, Y. Wang, D. Xue, D. Li, *Dalton Trans.* **2020**, *49*, 711-718.
